# Occupational health outcomes among migrant and non-migrant construction workers: A systematic review and narrative synthesis

**DOI:** 10.1371/journal.pgph.0007624

**Published:** 2026-11-13

**Authors:** Jahnavi Prabhala, Sonica Minhas, Ethan Levine, Caroline Park, Samuel Caplan, Riley Mitchell, Cathy Zimmerman, Xichen Wang, Jason Rydberg, Joelle Mak, Stephanie Banning, Vijeta Bhambhani, Diya Jayram, Ana Maria Buller, Hanni Stoklosa

**Affiliations:** 1 Harvard Medical School, Harvard University, Boston, Massachusetts, United States of America; 2 Department of Social Policy and Intervention, University of Oxford, Oxford, United Kingdom; 3 Coalition to Abolish Slavery & Trafficking (CAST), Los Angeles, California, United States of America; 4 Department of Sociology and Anthropology, Stockton University, Galloway, New Jersey, United States of America; 5 HEAL Trafficking, Long Beach, California, United States of America; 6 Tufts University School of Medicine, Tufts University, Boston, Massachusetts, United States of America; 7 Department of Global Health and Development, London School of Hygiene and Tropical Medicine, London, United Kingdom; 8 School of Criminology and Justice Studies, University of Massachusetts Lowell, Lowell, Massachusetts, United States of America; 9 Center for Program Evaluation, School of Criminology and Justice Studies, University of Massachusetts Lowell, Lowell, Massachusetts, United States of America; 10 Department of Health Services Research and Policy, London School of Hygiene and Tropical Medicine, London, United Kingdom; 11 Department of Medicine, Beth Israel Deaconess Medical Center, Boston, Massachusetts, United States of America; 12 Harvard T.H. Chan School of Public Health, Harvard University, Boston, Massachusetts, United States of America; 13 Brigham and Women’s Hospital, Boston, Massachusetts, United States of America; 14 University of Miami Miller School of Medicine, Miami, Florida, United States of America; 15 Department of Emergency Medicine, Brigham and Women's Hospital, Boston, Massachusetts, United States of America; Directorate of Factories, INDIA

## Abstract

Construction remains one of the world’s most hazardous industries, with migrants comprising a substantial part of its workforce. Little evidence exists on occupational health disparities between migrant and non-migrant construction workers. This review compares occupational health outcomes between these two groups to inform equitable worker protection strategies. Following PRISMA guidelines, we systematically searched fourteen electronic databases and gray literature for studies published from January 1990 to May 2025. Eligible studies reported quantitative occupational health outcomes for both groups. Study quality was appraised using adapted Joanna Briggs Institute criteria and results were narratively synthesized. Thirty-eight studies across fifteen high-, upper-middle, and lower-middle-income countries were included, totaling 55,615 adverse health outcomes among migrant construction workers and 566,643 among non-migrant construction workers. Most studies were of low to moderate quality and were primarily conducted in high-income settings. Migrant construction workers experienced higher rates of fatal and general occupational injury than non-migrants. While migrants had excess risk for fatal falls, traumatic brain injury, heat-related mortality, and ocular trauma, they experienced lower all-cause morbidity and mortality. Patterns of non-fatal injuries, musculoskeletal disorders, mental health problems, and cardiovascular and metabolic disease were mixed. Occupational and structural factors significantly associated with morbidity and/or mortality among migrants included fewer years of employment and work experience, type of work, limited safety training and briefing attendance, and lower educational attainment. Associated socio-demographic and cultural factors included poor language proficiency, ethnicity, gender, risk-taking attitudes, and low awareness of workplace hazards; the role of age varied across studies. Migrant status is a significant determinant of occupational health in the construction sector. Strengthening labor protections for migrant workers and providing multilingual safety training are likely to reduce harm. Disaggregating occupational safety and health surveillance data by migrant status will further strengthen the evidence needed to protect the most at-risk construction workers. **Registration:** PROSPERO CRD42014009154.

## Introduction

Construction is among the most dangerous occupational sectors, accounting for the largest number of injuries and fatalities, with an estimated 30% of all occupational fatalities worldwide [1,2]. Construction workers face diverse physical, chemical, biological, and psychosocial hazards that contribute to adverse health outcomes like silicosis, asbestosis, traumatic brain injury, fractures, tuberculosis, histoplasmosis, depression, and premature death [3]. Global attention to occupational risks among migrant workers increased during the COVID-19 pandemic and amidst large-scale international sporting events, such as the Olympics and the FIFA World Cup [4]. Preparations for the Qatar 2022 World Cup triggered a surge in migrant labor and provoked international scrutiny following reports of hazardous working conditions, exemptions from COVID-19 lockdown measures, and deaths of thousands of workers [5–7].

Labor migration is a growing phenomenon, shaped by globalization, urbanization, demographic shifts, and climate change, which influence the supply and demand of labor [8]. As a major global employer, the construction industry has become increasingly reliant on migrant labor to address labor shortages and reduce costs [9]. According to the International Labour Organization (ILO), migrants comprise 30–40% of the global construction workforce, and as much as 80–90% in Gulf Cooperation Council countries [10]. In higher-income countries, including the United States (US), migrants account for 20–25% of the construction workforce, with higher shares in rapidly urbanizing regions [10–12].

Construction workers encounter numerous occupational health and safety risks. For example, they are exposed to occupational risks such as high temperatures, heavy lifting, hazardous chemicals, working at high elevations, and operating large machinery [13,14]. For migrants, these work-related hazards are often created or exacerbated by exposures throughout the migration journey, and the socio-economic and structural inequalities that affect migrant workers at their destination. Migrant health patterns are dynamic and heterogeneous, often reflecting exposures along the migration trajectory, from pre-departure risks to conditions in the receiving country [15]. While some individuals may arrive in better health than the native population, a phenomenon known as the ‘healthy immigrant effect,’ those employed in low-skill, low-wage industries are disproportionately burdened with intersecting social, economic and health barriers. These risks include exploitation during recruitment, legal concerns, poor living conditions, limited healthcare access and social protection, and language barriers, which can collectively erode any initial advantage [13,16,17]. Recruitment exploitation in the construction industry can range from deceptive practices to labor trafficking and forced labor, the latter defined by the ILO as “all work or service which is exacted from any person under the threat of a penalty and for which the person has not offered himself or herself voluntarily” [18]. Coercive conditions can increase workers’ risks of injury and fatality by limiting their ability to refuse, report, or leave unsafe work. Further, these structural disadvantages can be reinforced by anti-immigration sentiment and hostile policy environments, which are increasingly recognized as detrimental to migrants’ mental health [19,20].

Despite the construction sector's substantial burden of morbidity and mortality, and its reliance on migrant labor, there is limited understanding of how health outcomes differ between migrant and non-migrant workers in the sector. Prior reviews have examined these differences across all occupational sectors, but none have focused on construction specifically [21–24]. As immigration policies, labor protections, and workers’ rights vary considerably between countries, a global review can elucidate consistent patterns of vulnerability and the structural factors influencing migrant construction worker health. Amid accelerating migration patterns worldwide and rapid urbanization, global evidence can help guide targeted interventions and policies that support the health and human rights of migrant construction workers. This review aims to: (1) examine differences in occupational health outcomes between migrant and non-migrant construction workers; (2) synthesize evidence on common risk and protective factors affecting migrant construction workers’ health; and (3) highlight knowledge gaps in migrant construction worker health to guide future research.

## Methods

This review follows the Preferred Reporting Items for Systematic Reviews and Meta-Analyses (PRISMA) guidelines [25] and is registered on PROSPERO (CRD42014009154). Study selection followed PRISMA standards (Fig 1, Results section).

**Fig 1:**
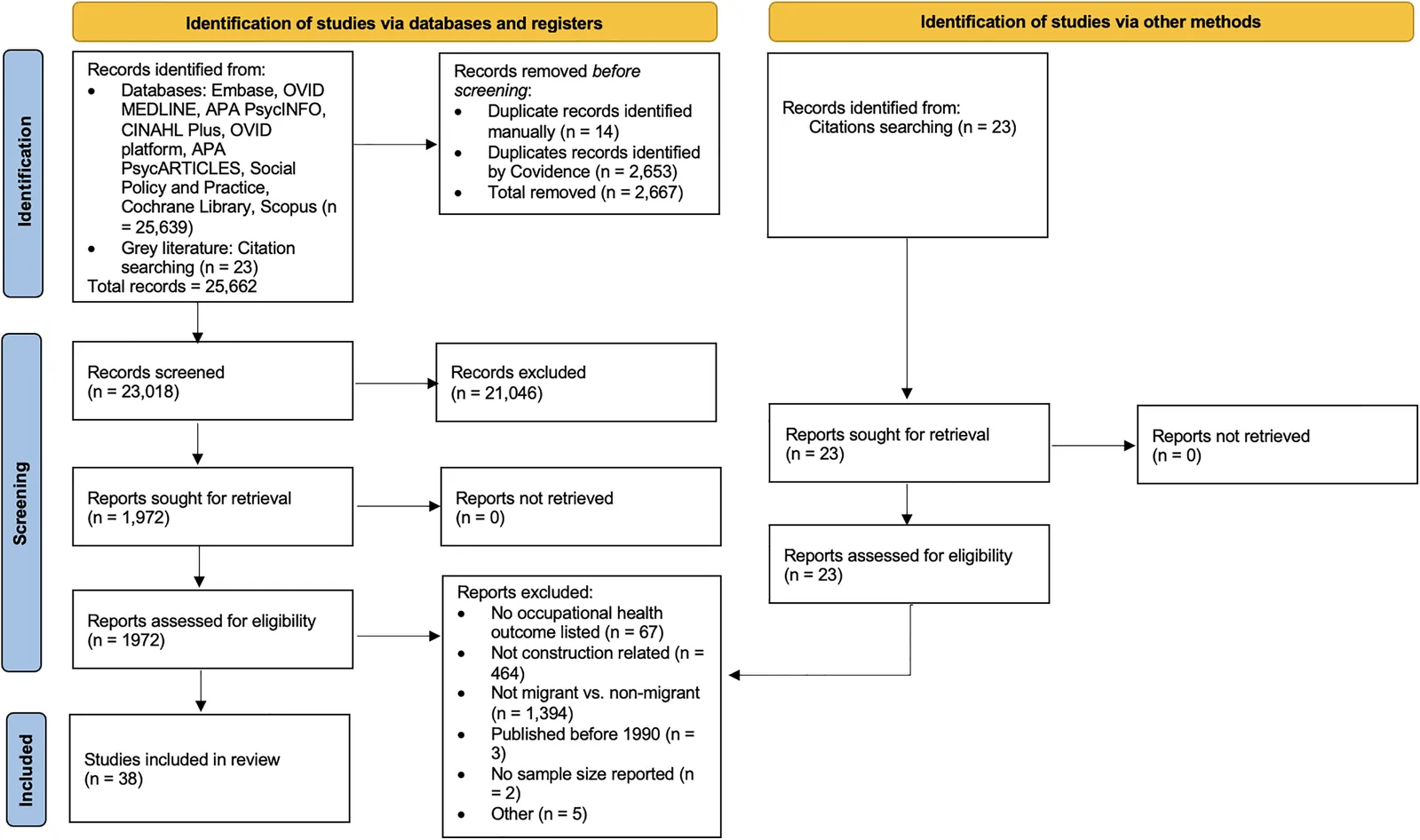
PRISMA diagram.

### Search strategy and eligibility criteria

We systematically searched fourteen electronic databases and gray literature sources to identify studies reporting on occupational health of migrant and non-migrant construction workers. The following databases were searched: The Cochrane Central Register of Controlled Trials (CENTRAL); MEDLINE; EMBASE; PsycINFO; Scopus; Web of Science; Google Scholar; Popline; and OSH-ROM (including NIOSHTIC, CISDOC). Gray literature sources included were: the ILO; Human Rights Watch (HRW); Veritas; Soros; and Le Strada International.

The search strategy was developed using the conceptual framework outlined in Fig 2, combining terms related to *‘construction worker’, ‘migrant worker’, ‘labor trafficking’, ‘occupational exposures’* and *‘negative health outcomes’* with Boolean operators [26]. Given the profound evolution of construction technologies and equipment since the early 1990s, the search was limited to studies published between January 1^st^, 1990 and May 15^th^, 2025.

**Fig 2:**
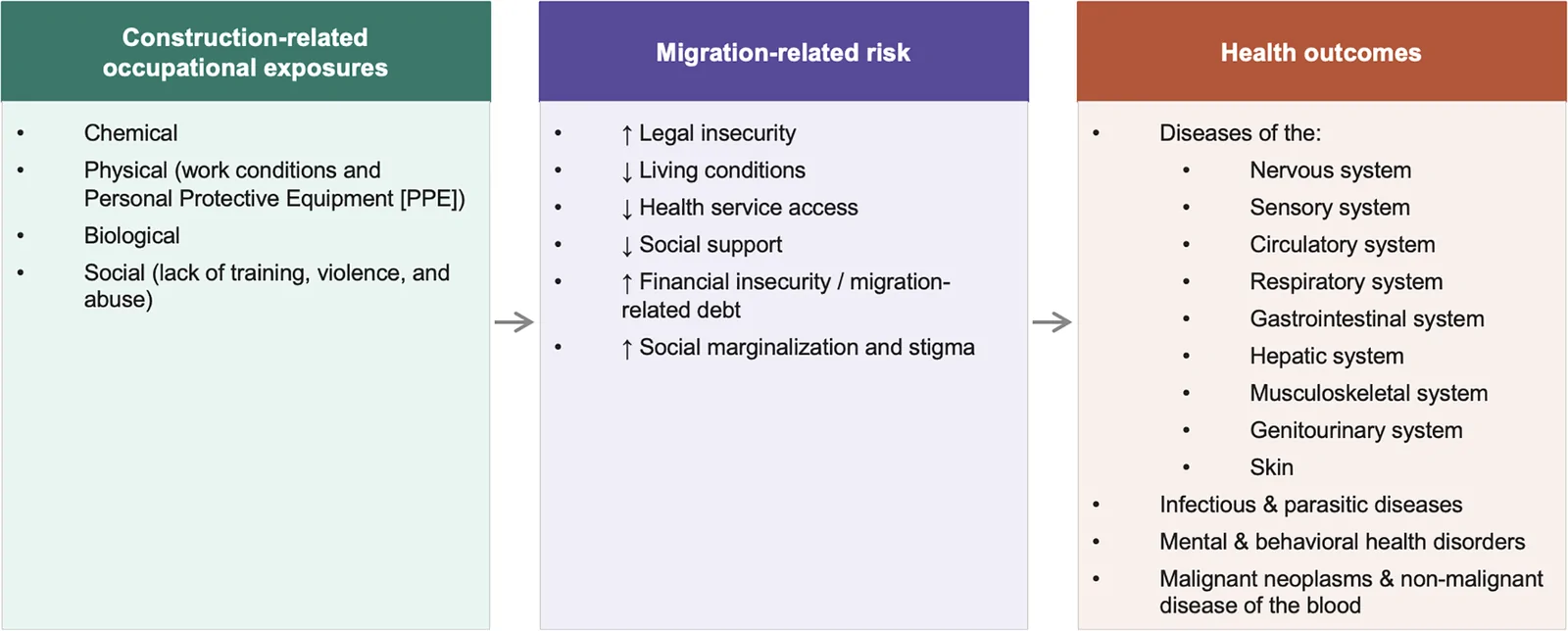
Conceptual framework ofoccupational exposures, migration-related risks, and health outcomes among migrant construction workers [26].

We screened the reference lists of all included studies and conducted forward and backward citation tracking to identify additional studies not captured by our initial search. We also contacted study authors and experts to identify other potentially relevant studies. S1 Text and S2 Text provide additional details regarding the search strategy and definitions of the key search terms.

Studies were included if they (1) included adult or child migrant and non-migrant construction workers; and (2) reported occupational health outcomes related to construction work for both migrant and non-migrant workers. No restrictions were placed on language, geographical region, or methodological approach for evaluating outcomes. Studies were excluded if they lacked data on sample size or an equivalent measure (e.g., person-years). S3 Text outlines the full inclusion and exclusion criteria.

### Screening and data extraction

Title-abstracts and full-texts were double-screened independently by two of eight authors (JP, CP, RM, SB, DJ, HS, VB, AMB) using Covidence. Disagreements were resolved by discussion between the two reviewers, with a third reviewer consulted if consensus was not reached.

The following data were extracted from included studies: study authors, study design, year of study, sample characteristics, number of participants, type of construction work, definitions of non-migrant and migrant, occupational exposures, health outcomes, and additional mediator or moderator variables. Where data were not reported or not in a usable format, study authors were contacted directly to obtain the data. Where authors did not respond or data remained unavailable, studies were retained in the narrative synthesis if they continued to meet the inclusion criteria. For example, studies reporting prevalence data without effect measures were still included in our review for descriptive statistics purposes only.

### Quality appraisal

All included studies were evaluated for quality of reporting using criteria adapted from the Joanna Briggs Institute (JBI) Checklist for Prevalence Studies, the Checklist for Analytical Cross-Sectional Studies, and the Checklist for Cohort Studies. The adapted checklist assigned a maximum of 16 points for prevalence studies and 20 points for comparative studies. Full scoring criteria are provided in S1 Table. Studies scoring ≥ 80% were rated high quality, 50–79% moderate quality, and <50% low quality. JP and SC independently conducted appraisal, resolving discrepancies through discussion; any unresolved discrepancies were reconciled by HS.

### Data analysis

The data abstraction and synthesis process was guided by the conceptual framework outlined in Fig 2. Data extraction was conducted in two phases by independent reviewer pairs (JM and HS; JP and SC), with HS serving as arbitrator to resolve discrepancies. The data categorization structure was developed by JP and reviewed for accuracy and consistency by CP and SC. Study findings were extracted and organized within this framework according to (1) negative health outcomes reported in each study, (2) mediators and moderators explicitly measured with supporting statistics, and (3) factors mentioned as potential mediators or moderators even if not directly analyzed. This framework allowed for systematic identification and comparison of relationships across studies. All data were recorded in Microsoft Excel to conduct a thematic analysis. Descriptive analyses were then conducted to summarize study and sample characteristics (e.g., country, age range, type of construction work, migrant and non-migrant status, sample size) and measurement approaches for health outcomes (e.g., prevalence, odds ratios, rates). Data were extracted directly from all included studies (n = 38). For 14 studies, we calculated prevalence estimates (n = 7), odds ratios and corresponding 95% confidence intervals (n = 7), and sample sizes (n = 4) in Excel. Meta-analysis was not possible due to variability in effect measures reported across included studies.

## Results

### Screening results

The study selection process is outlined in Fig 1. Initially, 25,662 records were identified, of which 2,667 were duplicates. 1,972 full-texts were reviewed for eligibility, of which 38 met the inclusion criteria. Primary reasons for exclusion included studies that examined migrant workers without a clear focus on the construction sector, those that lacked a direct comparison between migrant and non-migrant construction workers, and studies whose target outcomes did not correspond with occupational health outcomes.

### Study characteristics

The included studies were from high-income (US, Ireland, Germany, Switzerland, Italy, Netherlands, Israel, Qatar, South Korea, Taiwan, Australia), upper-middle-income (Iran, China, Thailand) and lower-middle-income (Lebanon) countries, according to World Bank classification [27].

All studies were quantitative and observational, comparing occupational health outcomes in migrant construction workers to a non-migrant worker reference population. More than half (21, 55%) utilized national or state-level datasets [28-48], while the remainder drew on primary data sources, including occupational, hospital, forensic, region- or company-specific datasets [49-65].

Across the included studies, there were 55,615 morbidity and mortality events recorded among migrant construction workers and 566,643 among non-migrant construction workers. Twenty-three studies reported morbidity outcomes, while 20 reported on mortality, with some reporting multiple morbidity and/or mortality outcomes.

The overall quality of studies was low to moderate, highlighting the need for improved methodological rigor in this field. Sixteen studies (42%) were rated as low quality (score<50%), 19 (50%) as moderate quality (score 50–79%), and 3 (8%) as high quality (score ≥ 80%). Common reasons for low study quality were missing effect sizes, non-random sampling methods, and insufficient information on response rates and determination of migration/nativity status. A detailed summary of study characteristics is available in Table 1.

**Table 1:** Study characteristics.

Title	Author, Year	Country	Years of Study	Prevalence vs. Comparative	Study Design (Sampling Method)	Study Population	Outcomes of Interest	Sample			Definition of Migrant	Quality Score
								Total Sample of Construction Workers†	Reference Non-Migrant Population	Migrant Population		
**All-cause and cause specific mortality in a cohort of 20,000 construction workers; results from a 10 year follow up**	Arndt, 2004	Germany	1986-2000	Prevalence	Retrospective cohort (purposive)	Male construction workers (plumbers, carpenters, painters, plasterers, bricklayers, and unskilled workers/laborers) aged 25–64 in the German construction industry who received routine voluntary occupational health examinations at the Institution for Statutory Accident Insurance and Prevention in Baden-Württemberg, Southern Germany between 1986 and 1992 and were followed up until 1999–2000.	All deaths, mortality due to Cancer, mortality due to Circulatory systems, mortality due to respiratory system, mortality due to digestive system, mortality due to Injury and poisoning, mortality due to all non-transport accidents, mortality due to falls, mortality due to struck by falling objects	N^1^ = 19,827 construction workers (plumbers, carpenters, painters, plasterers, bricklayers, and unskilled workers/laborers)	14,725 Germans	5,102 Non- Germans (1814 Former Yugoslavians, 1359 Italians, 1277 Turkish, 652 Other)	Non-German nationality (Former Yugoslavia, Italian, Turkish, Other)	68.75%
**Construction work and risk of occupational disability: a ten year follow up of 14,474 male workers**	Arndt, 2005	Germany	1986-2000	Prevalence	Retrospective cohort (purposive)	Male construction workers (plumbers, carpenters, painters, plasterers, bricklayers, and unskilled workers/laborers) aged 25–64 in the German construction industry who received routine voluntary occupational health examinations at the Institution for Statutory Accident Insurance and Prevention in Baden-Württemberg, Southern Germany between 1986 and 1992 and were followed up until 1999–2000.	All-cause disability, disability due to musculoskeletal disorders, disability due to accidents	N^2^ = 14,474 construction workers (plumbers, carpenters, painters, plasterers, bricklayers, and unskilled workers/laborers) in general work force and blue collar workforce	11,974 Germans	2,500 Non-Germans (737 Former Yugoslavians, 674 Italians, 655 Turkish, 434 Other)	Non-German nationality (Former Yugoslavia, Italian, Turkish, Other)	68.75%
**Body Mass Index and Premature Mortality in Physically Heavily Working Men—A Ten-Year Follow-Up of 20,000 Construction Workers**	Arndt, 2007	Germany	1986-1992	Prevalence	Retrospective cohort (purposive)	Male bricklayers, painters, laborers, plumbers, carpenters, and plasterers aged 25–64 in the German construction industry who received routine voluntary occupational health surveillance in Württemberg, Southern Germany, between 1986 and 1992.	BMI [Intermediate/Poor BMI >=25 kg/m2]	N‡ = 19513 construction workers	14440 German ‡	1756 Former Yugoslavian, 1366 Italian, 1366 Turkish, 585 Other ‡	Non-German nationality (Former Yugoslavia, Italian, Turkish, Other)	43.75%
**Hispanic/Latino fatal occupational injury rates**	Byler, 2013	USA	2006-2008	Prevalence	Retrospective cross-sectional (population-based)	All workers in the U.S. who sustained an occupational fatality 2006–2008 (based on data from the U.S. Census of Fatal Occupational Injuries and Current Population Survey).	Fatal injuries	N‡ = 817 construction trade workers (carpenter, construction laborers, roofers)	195 Native-born Hispanic/Latino workers	622 Foreign-born Hispanic/Latino workers	Foreign-born	18.75%
**Differences in patterns of mortality between foreign-born and native-born workers due to fatal occupational injury in the USA from 2003 to 2010**	Byler, 2018	USA	2003-2010	Comparative	Retrospective cross-sectional (population-based)	All native and foreign-born U.S. construction workers who suffered an occupational fatality between 2003 and 2010, identified through the U.S. Census of Fatal Occupational Injuries.	Occupational fatalities/mortalities, fatal injury risk	N = 11,012 construction and maintenance workers	8698 US-born	2314 Foreign-born	Foreign-born	70%
**Ethnicity, work-related stress and subjective reports of health by migrant workers: A multi-dimensional model**	Capasso, 2018	Italy	2012-2014	Comparative	Retrospective cross-sectional (multi-stage & convenience sampling)	Eastern European care workers, Moroccan factory workers, Ghanaian construction masons, and Italian counterparts in similar industries aged 25–45 years recruited through migrant associations in Southern Italy.	Interpersonal disorders, anxious-depressive disorders, general health	N = 300 masons	100 Italian	200 Ghanaian	First-generation immigrants	60%
**Fatal and non-fatal occupational injuries and diseases among migrant and native workers in South Korea**	Cha, 2014	South Korea	2005-2007	Comparative	Retrospective cross-sectional (population- based)	All migrant workers in Korea compensated for fatal and non-fatal occupational injuries and diseases by the National Occupational Injury Compensation Insurance between 2005 and 2007 (from Korea Workers Compensation and Welfare Center records), and all Korean workers with occupational injuries and diseases reported by the Ministry of Labor during the same period.	Work-related fatal injury, work-related non-fatal injury, work-related fatal disease, and work-related non-fatal disease from 2005-2007	N = 7,597,171 construction workers (sum of 2005–2007 annual workforces)	7,562,842 Korean	34,329 Migrant	All immigrant workers in Korea	70%
**Cardiovascular health of construction workers in Hong Kong: A cross-sectional study**	Chung, 2018	China	2014-2016	Prevalence	Retrospective cross-sectional (convenience sampling)	Registered construction workers in Hong Kong recruited through convenience sampling from 46 construction sites (across the New Territories, Kowloon, and Hong Kong Island) who participated in the “Pilot Medical Examination Scheme” between 2014 and 2016.	Weight Status [Intermediate/Poor (BMI>=23 kg/m2], Blood Cholesterol [Intermediate/Poor (>=5.2 mmol/L or 200 mg/dL)], Blood Pressure [Intermediate/Poor (>=120mmHg/ 80mmHg)], Blood Glucose [Intermediate/Poor (>=5.6 mmol/L or 100 mg/dL)]	N = 626 construction workers	526 Hong-Kong Born Chinese	77 Mainland-born Chinese, 23 Minority (from Nepal, Pakistan, or other countries)	Ethnicity (Chinese: born in Hong Kong or have lived in Hong Kong for more than 7 years, new Chinese migrants: from Mainland, Minority).	55%
**Construction-related eye injuries in Irish nationals and non-nationals: attitudes and strategies for prevention**	Connell, 2007	Ireland	2006	Comparative	Cross-sectional case-series (convenience)	All construction workers presenting with construction-related eye injuries at Royal Victoria Eye and Ear Hospital, Accident and Emergency Department during a 2-month study period in 2006.	Construction-related ocular injuries	N = 155 construction workers	80 Irish	45 Polish, 5 Romanian, 16 Lithuanian, 9 Other (non-Irish)	Non-national workers (Polish, Romanian, Lithuanian, Other)	18.75%
**Role of migrant factors in work-related fatalities in Australia**	Corvalan, 1994	Australia	1982-1984	Comparative	Retrospective cross-sectional (population-based)	All employed civilian workers in Australia who died from work-related injuries between 1982 and 1984, as identified through coronial records and classified by nationality and language group (based on ICD-9 external cause codes). Additional data provided via the Australian Labor Force Survey and Bureau of Statistics.	Work-related fatalities	N = 182 construction workers	111 Australian-born	26 English-speaking migrants, 45 Non-English speaking migrants	Country of birth (non-English speaking countries, English speaking countries - defined persons from English-speaking countries as those born in the United Kingdom and Ireland, the United States, Canada, New Zealand, and South Africa)	56.25%
**Characterization of Foreign-Born vs. Native-Born Worker Fatalities in Kentucky, 2001–2014**	Cruz, 2018	USA	2001-2014	Comparative	Retrospective cross-sectional (population-based)	All workers who experienced a work-related fatal injury in the state of Kentucky and were captured in the Kentucky Fatality Assessment and Control Evaluation (FACE) program data, funded by National Institute for Occupational Safety and Health.	Worker fatality	N = 225 construction industry workers	202 US-born	23 Foreign-born	Foreign-born	40%
**Occupational injuries in foreign-national workers presenting to St James Hospital Plastic Surgery Service**	Davidson, 2009	Ireland	2006-2007	Prevalence	Retrospective cross-sectional (convenience; hospital-based case series)	All workers presenting with work-related injuries to the Plastic Surgery service at St. James’s Hospital, Dublin, from August 2006 to February 2007 (based on prospectively collected clinical questionnaires completed by attending doctors).	Work-related injuries	N = 82 construction workers	52 Irish	30 Foreign-national	Foreign-Nationality	25%
**Fatal falls among Hispanic construction workers**	Dong, 2009	USA	1992-2006	Prevalence	Retrospective cross-sectional (population-based)	All construction workers aged 16 years and older in the U.S. who died from occupational fall-related injuries between 1992 and 2006 (based on data from the Census of Fatal Occupational Injuries and the Current Population Survey).	Fatal falls	N = 1641 construction workers	1150 US-born (103 Hispanic, US-born)	491 Foreign-born (396 Hispanic, Foreign-born)	Foreign-born (Mexico, Central America, South America, Caribbean, Other)	70%
**Fatal falls from roofs among U.S. construction workers**	Dong, 2013	USA	1992-2009	Prevalence	Retrospective cross-sectional (population-based)	All U.S. construction workers aged 16 years and older who died from occupational fall-related injuries off roofs between 1992 and 2009 (based on data from the Census of Fatal Occupational Injuries and the Current Population Survey).	Fatal falls from roof, other fatal falls, all fatalities	N = 8123 construction workers	6255 US-born	1868 Foreign-born	Foreign-born	50%
**Fatal falls in the U.S. residential construction industry**	Dong, 2014	USA	2003-2010	Prevalence	Retrospective cross-sectional (population-based)	All U.S. construction workers who died from occupational fall-related injuries between 2003 and 2010, identified through the Census of Fatal Occupational Injuries and Current Employment Statistics, categorized as residential or non-residential construction sector according to the North American Industry Classification System.	Fatal falls	N = 2986 construction workers	2281 US-born	705 Hispanic, Foreign-born	Foreign-born	43.75%
**Long-term health outcomes of work-related injuries among construction workers-findings from the National Longitudinal Survey of Youth**	Dong, 2015	USA	1988-2006	Prevalence	Prospective cohort (population-based)	Blue-collar construction workers who completed the U.S. National Longitudinal Survey of Youth 1979 cohort (NLSY79), followed prospectively from 1988 to 2006, with health outcomes measured at age 40 (1998–2006) and stratified by prior work-related injury status.	Worker injury	N‡ = 1435 Blue-collar construction workers	1385 US-born‡	50 Foreign-born‡	Foreign-born	37.50%
**Heat-related deaths among construction workers in the United States**	Dong, 2019	USA	1992-2016	Prevalence	Retrospective cross-sectional (population-based)	All U.S.-based construction workers aged 16 years or older who died due to occupational heat-related causes between 1992 and 2016, as recorded in the Census of Fatal Occupational Injuries, with employment denominator estimates from the Current Population Survey (CPS).	Heat-related death	N = 82 construction workers	56 US-born	21 Mexico-born, 5 Born in “other” country	Birthplace (the U.S. or other countries)	50%
**Ethnic difference in disability risk between Dutch and Turkish scaffolders**	Elders, 2004	Netherlands	1981-2000	Comparative	Retrospective cross-sectional (purposive)	All Dutch and Turkish male scaffolders who became disabled while employed by a large scaffolding company in the Netherlands between 1981 and 2000 (based on occupational health service records).	Back disorders, other musculoskeletal disorders, accidents, psychiatric disease, cardiovascular disease, other disorders, unknown disorders	N = 256 scaffolders	125 Dutch	131 Turkish	Turkish workers born in Turkey or with at least one parent born in Turkey	50%
**Traumatic Occupational injuries in Hispanic and foreign born workers**	Forst, 2010	USA	2000-2007^3^	Comparative	Retrospective cross-sectional (convenience-sampling)	All patients with work-related injuries admitted to a large urban trauma center in a metropolitan area of Illinois (USA) from 2000 to 2007 via EMS services.	Occupational injury	N = 22 construction laborers	9 US-born	13 Foreign-born	Foreign-born	25%
**782 consecutive construction work accidents: who is at risk?**	Frickmann, 2012	Switzerland	2001-2011	Prevalence	Retrospective cross-sectional (convenience)	All construction workers presenting to the University Emergency Department in Berne, Switzerland, with work-related injuries from 2001 to 2011 (based on hospital electronic medical records).	Occupational accidents	N = 782 construction workers	263 Swiss	519 Non-Swiss	Non-national workers (Non-Swiss; citizenship)	37.50%
**Migrant workers in Italy: an analysis of injury risk taking into account occupational characteristics and job tenure**	Giraudo, 2017	Italy	2000-2005	Comparative	Retrospective cross-sectional (population-based)	All private-sector native Italian and migrant male workers employed between 2000 and 2005, identified from the Work History Italian Panel (WHIP)-Salute integrated database.	Occupational injuries	N^4^ = 197,058 construction person years	137,333 person years HIC (individuals born in high-income countries)	59,725 person years of workers born in SMPC, 24,912 person years of which born in Morocco	Workers born in countries with strong migratory pressure (SMPC: Africa; Asia excluding Israel, South Korea and Japan; Latin America; Central and Eastern Europe) and Moroco	80%
**Differences in work injury risk between immigrants and natives: changes since the economic recession in Italy.**	Giraudo, 2019	Italy	2005-2010	Comparative	Retrospective cross-sectional (population-based)	All male blue-collar and apprentice workers aged 16–55 employed in the Italian metalworking and construction sectors in 2005 or 2010, with active employment contracts recorded in the WHIP-Salute integrated database.	Occupational injuries from 2005-2010	N^5^ = 56,843 construction person years in 2005; N = 52,999 in 2010	45,661 person years HIC (individuals born in high-income countries) in 2005; 40,142 in 2010	11,182 person years, SMPC-born workers in 2005; 12,857 in 2010	Workers born in countries with strong migratory pressure (SMPC: Africa; Middle East and Asia excluding Israel,South Korea, and Japan; Latin America; Central and Eastern Europe)	80%
**Prevalence of occupational accidents and factors associated with deaths and disabilities in the shipbuilding industry: Comparisons of novice and skilled workers.**	Jeong, 2021	South Korea	2016-2017	Prevalence	Retrospective cross-sectional (population-based)	All workers in the shipbuilding industry in the Republic of Korea whose occupational injuries (excluding commuting and outside activity accidents) were officially approved for workers’ compensation in 2016–2017, as recorded in Ministry of Labor administrative data.	Occupational injury	N = 2069 shipbuilders	1944 Korean	125 Foreigner	Nationality (Korean vs. Foreigner)	43.75%
**Epidemiology of occupational injuries in construction workers between 2009 and 2018 in South Korea**	Jung, 2023	South Korea	2009-2018	Prevalence	Retrospective cross-sectional (population-based)	All construction workers in South Korea with accepted workers’ compensation claims for occupational injuries between 2009 and 2018, based on data collected from the Korea Workers’ Compensation & Welfare Service (KCOMWEL).	Total injury claims, non-fatal injury claims (male, female), fatal injury claims (male, female)	N = 158,947 construction workers	148,404 South Korean domestic workers	10,543 foreign workers	Foreign workers	50%
**Variations in suicide rates among Australian male construction workers by country of birth**	King, 2024	Australia	2006-2020	Comparative	Retrospective cross-sectional (population- based)	All male construction workers and non-construction workers in Australia who died by suicide between 2006 and 2020, based on data from the National Coronial Information System (NCIS).	Suicide death	N = 3093 construction workers	2618 Australian	251 English-speaking country of origin, 103 Non-English-speaking European country of origin, 121 Other countries	Country of Origin: English-speaking countries, Non-English speaking countries European countries, Other countries	65%
**Fatal traumatic brain injuries in the construction industry, 2003–2010**	Konda, 2016	USA	2003-2010	Prevalence	Retrospective cross-sectional (population-based)	All wage and salary construction workers aged 16 years and older in the United States who sustained fatal traumatic brain injuries (TBIs) between 2003 and 2010 (based on data from the U.S. Census of Fatal Occupational Injuries (CFOI) and Current Population Survey).	Fatal traumatic brain injury (TBIs)	N = 2210 construction workers	1560 Native US-born	650 Foreign-born	The foreign born are persons who reside in the United States but who were born outside the country or one of its outlying areas to parents who were not US citizens. The native born are persons who were born in the United States or one of US outlying areas or born abroad to born abroad to US citizen-parents.	62.50%
**Comparison of Safety Perception between Foreign and Local Workers in the Construction Industry in Republic of Korea**	Korkmaz, 2018	South Korea	2016	Comparative	Cross-sectional (convenience)	All Korean and foreign construction workers who completed a multilingual safety perception questionnaire at selected construction sites in Seoul, Pyeongtaek, and Gyeonggi, South Korea.	Workplace accidents	N = 891 construction workers	751 Korean	140 Foreign	Foreign workers (Nationality: Korea, China, Vietnam, Kazakhstan, The Philippines, Bangladesh, Thailand, Kyrgyzstan, Pakistan, India)	50%
**Work-related injuries in Lebanon: Does Nationality Make a Difference?**	Nuwayhid, 2003	Lebanon	1998	Comparative	Retrospective cross-sectional (purposive; insurance claims)	All injured workers in Lebanon covered by work-related accident insurance in 1998 at five major insurance companies recorded under MedNet, a third party administrator.	Work-related injuries: hand, feet, eyes, upper body, lower body, multiple parts, information missing	N = 1,623 construction industry workers	561 Lebanese	861 Syrians, 201 Other nationalities	Nationality (categorized as Lebanese, Syrian, and other nationalities).	50%
**Risk Assessment among Thai and Foreign Workers in Construction Companies**	Pawthaisong, 2018	Thailand	2015-2016	Comparative	Mixed-methods cross-sectional (population-based, purposive)	All construction workers (Thai and foreign) who were recorded to have an occupational injury at three selected construction companies in Thailand from 2015 to 2016. Additional occupational injury data retrieved from Thailand's Workmen's Compensation Fund in 2016.	Death, disability, minor injury, accident number from 2012 and 2013	N = 8806 construction workers including Engineer, Accountant, Management, Craftsman and Worker (year 2012), N = 8722 (year 2013)	4147 Thai (year 2012), 4104 Thai (year 2013)	4659 Foreign (year 2012), 4618 Foreign (year 2013)	Foreign workers (non-nationals)	30%
**Risk Perception and Ethnic Background in Construction Workers: Results of a Cross-sectional Study in a Group of Trainees of a Vocational School in Italy**	Ricci, 2021	Italy	Unknown	Comparative	Cross-sectional (purposive)	All Italian and migrant construction workers aged 16–65 who voluntarily completed a questionnaire during occupational health and safety training at vocational Building Schools (Scuole Edili) affiliated with FORMEDIL in Emilia-Romagna and Sardinia, Italy.	Previous personal experience of occupational injury	N = 562 construction workers	407 Italians	22 North African, 80 Eastern Europe, 53 Balkans	Region of Origin: North Africa (Algeria, Egypt, Morocco, Tunisia), Eastern Europe (Lithuania, Poland, Moldova, and Romania), Balkans (Albania, Croatia, Kosovo, Serbia)	55%
**Epidemiology of Occupational Electrical Injuries**	Salehi, 2022	Iran	2017-2018	Prevalence	Retrospective cross-sectional (purposive)	All patients with electrical burns referred to Shahid Motahari Educational and Medical Center in Tehran, Iran, between April 2017 and March 2018, identified through hospital medical records.	Electrical injuries/burns	N = 66 construction workers	49 Iranians	17 Non-Iranians	Non-Iranians (nationality)	25%
**Work-related injuries among immigrant worker in Italy**	Salvatore, 2012	Italy	2007	Comparative	Retrospective cross-sectional (two-stage sampling)	Immigrant and native Italian workers (aged ≥15) who responded to the 2nd quarter 2007 Labour Force Survey conducted by Italy’s National Institute of Statistics (ISTAT).	Work-related injuries	N = 4316 construction workers (skilled and unskilled)	3886 Italian	430 Immigrant	Country of citizenship	85%
**Safety is Seguridad: A workshop summary**	Scrimshaw, 2003	USA	1995-2000	Prevalence	Retrospective cross-sectional (population-based)	All construction workers in the U.S. who sustained an occupational fatality from 1995 to 2000 (based on data from the Census of Fatal Occupational Injuries).	Fatal work injuries, fatal occupational injuries	N‡ = 1153 construction workers (construction laborers, roofers, carpenters/apprentices)	397 Native-born, Hispanic‡	756 Foreign-born, Hispanic‡	Foreign-born	25%
**Examining Occupational Health and Safety Disparities Using National Data: A Cause for Continuing Concern**	Steege, 2014	USA	2005-2009	Prevalence	Retrospective cross-sectional (population-based)	All workers in the U.S. who sustained an occupational fatality 2005–2009 (based on data from the U.S. Census of Fatal Occupational Injuries and Current Population Survey).	Fatal occupational injuries	N = 5674 construction industry workers	4336 Not foreign-born	1338 Foreign-born	Foreign-born	43.75%
**Epidemiology of workplace-related fall from height and cost of trauma care in Qatar**	Tuma, 2013	Qatar	2007-2008	Prevalence	Retrospective cross-sectional (purposive)	All adult male patients who sustained workplace-related falls and presented to Hamad General Hospital Trauma Center between 2007 and 2008 (based on records in the Hamad General Hospital Trauma Center Registry).	Work-related falls	N = 281 construction industry workers	8 Qatar nationals	273 Non-nationals	Non-national workers	18.75%
**Epidemiological study of occupational injuries among foreign and native workers in Taiwan**	Wu, 1997	Taiwan	1991-1993	Comparative	Retrospective cross-sectional (population-based)	All legally registered foreign workers in Taiwan from July 1991 to December 1993 with labor insurance records from the Bureau of Labor Insurance, and native Taiwanese labor-insured workers from the same industries in 1992.	Occupational injury	N = 15,201 foreign construction workers (Taiwanese N not reported)	Not reported^6^	15,201 Foreign	Foreign workers (excluding foreign workers who were in Taiwan for less than 1 year)	62.50%
**Occupational fatalities in Israel**	Yanai, 1999	Israel	1996-1998	Prevalence	Retrospective case-file reviews (purposive)	All fatally injured workers (local and foreign) undergoing forensic autopsy at the Israeli National Center of Forensic Medicine between 1996 and 1998 (identified from postmortem records and labor force data from the Ministry of Labor and Central Bureau of Statistics).	Fatal occupational accidents	N = 101 construction workers	38 Israeli	16 Palestinian, 47 Foreign	Nationality (Palestinian, foreign workers from the Far East - Thailand and China - and Eastern Europe - Romania, Ukraine, and Moldavia)	12.50%
**Work-related non-fatal injuries among foreign-born and US-born Workers: Findings from the U.S. National Health Interview Survey 1997–2005**	Zhang, 2009	USA	1997-2005	Comparative	Retrospective cross-sectional (population-based)	All foreign-born and native-born workers in the U.S. who were interviewed in the National Health Interview Survey (NHIS) between 1997 and 2005 (drawn from national household survey data using complex stratified sampling).	Work-related non-fatal injuries	N = 197 construction workers	174 US-born	23 Foreign-born	Foreign-born	70%

†Construction worker sample sizes only are reported in the review; included studies may have examined broader worker populations.

‡Sample sizes calculated by review authors based on percentages reported in included studies.

^1^Arndt, 2004: Baseline cohort comprised 19,943 workers; final study sample reported as 19,827 workers based on follow-up information.

^2^Arndt, 2005: Baseline cohort comprised 18,760 workers; final study sample reported as 14,474 workers based on follow-up information.

^3^Forst, 2010: Study period was 2000–2007; data extracted for this review are from 2003 only based on qualitative interviews with 22 construction workers.

^4^Giraudo, 2017: Sample size reported in person years; per the study, approximately 812,624 person years were observed during the study period, equivalent to 397,986 workers.

^5^Giraudo, 2019: Sample size reported in person years.

^6^Wu, 1997: The number of native Taiwanese construction workers was not reported in the original study; approximately 418,000 person years are implied by the reported injury count and incidence rate.

### Non-fatal injuries

Non-specific occupational injuries or accidents were the most commonly reported outcome (n = 17) [28,29,32,35–37,41–43,47,49,52,55,56,60,61,64]. Rates of non-specific or non-fatal injury or illness are listed in Table 2. Three studies explicitly examined non-fatal injuries [28,37,47] and one [43] reported on combined outcomes resulting in either death or disability.

**Table 2:** Rates of non-specific or non-fatal injury or illness among migrant and non-migrant construction workers.

Author, Year	Non-migrant group	Migrant group	Type of injury	Non-Migrant rate (95% CI)	Migrant rate (95% CI)	Unit of analysis (for Rate)
Cha, 2014	Korean	Migrant	Work-related non-fatal injury (2005)	74.99	356.6	per 10,000 workers
			Work-related non-fatal injury (2006)	68.99	192.56	per 10,000 workers
			Work-related non-fatal injury (2007)	63.79	850.4	per 10,000 workers
			Work-related non-fatal disease (2005)	1.38	7.64	per 10,000 workers
			Work-related non-fatal disease (2006)	2.84	5.56	per 10,000 workers
			Work-related non-fatal disease (2007)	3.35	26.9	per 10,000 workers
Giraudo, 2017	Italians; i.e., HIC: individuals born in high-income countries	SMPC: individuals born in countries with strong migratory pressure (SMPC: Africa; Asia excluding Israel, South Korea and Japan; Latin America; Central and Eastern Europe)	Occupational injuries	10.09 (9.56, 10.62)	11.21 (9.97, 12.44)	per 1000 person-years
		of which born in Morocco			14.33 (12.84, 15.82)	per 1000 person-years
Giraudo, 2019	Italians; i.e., HIC: individuals born in high-income countries	SMPC: individuals born in countries with strong migratory pressure (SMPC: Africa; Asia excluding Israel, South Korea and Japan; Latin America; Central and Eastern Europe).	Occupational injuries (2005)	13.34 (6.12, 20.56)	11.24 (9.47, 13.00)	per 1000 workers
			Occupational injuries (2010)	2.47 (1.85, 3.10)	9.46 (6.71, 12.21)	per 1000 workers
Pawthaisong, 2018	Thai workers	Foreign workers	Accident number^1^	23.15	64.78	per 1000 workers
			Occupational injuries (2016)^1^	14.47	31.18	per 1000 workers
Wu, 1997	Taiwanese	Foreign	Occupational injury^2^	8.04	5.33	per 1000 workers (‰)
	Taiwanese - male	Foreign - male		11.14	4.09	per 1000 workers (‰)
	Taiwanese - female	Foreign - female		3.47	760	per 1000 workers (‰)
Zhang, 2009	US-born	Foreign-born	Work-related non-fatal injuries	214 (183, 251)	104 (67, 163)	per 10,000 workers

^1^Pawthaisong, 2018: Occupational injuries data were extracted from the 2016 Thailand Workmen’s Compensation fund, whereas the Accident number data were based on primary data collected at three selected construction company sites.

^2^Wu, 1997: 1-year incidence rate, adjusted for age distribution.

Seven studies (Italy, Netherlands, South Korea, Taiwan) found that migrant construction workers, or subgroups of migrant workers, faced statistically higher risks of injuries and accidents compared to non-migrant workers [28,29,35,41–43,52]. In Taiwan, Wu et al. found that this excess was limited to female migrant workers [29]. In the Netherlands, Elders et al. [52] noted that Turkish scaffolders faced over twice the risk of disability compared to Dutch scaffolders (risk ratio [RR] 2.48, 95% CI 1.94-3.18), even after adjusting for age. In Italy, Giraudo et al. (2017) [41] reported that Moroccan migrants had an elevated injury risk compared to native-born workers in Italy (incidence rate ratio [IRR] 1.41, 95% CI 1.24-1.60; IRR for Italians 1). In contrast, migrants from countries with strong migratory pressure (defined by Giraudo et al. as countries from Africa, Asia, Latin America, Central and Eastern Europe, excluding Israel, South Korea, and Japan) did not differ significantly (IRR 1.10, 95% CI 0.98-1.23; IRR for Italians 1) [41].

Four studies (Switzerland, United States, Lebanon, Thailand) reported a higher prevalence of work-related injuries among migrants but did not include statistical comparisons [36,49,56,61].

Three studies discussed increased occupational hazards for migrants disaggregated by injury severity and outcome. In South Korea, Cha and Cho [28] identified elevated injury risk for non-fatal injury (crude RR for 2007 13.33, 95% CI 12.22-14.54) and non-fatal disease (crude RR for 2007 8.04, 95% CI 5.04-12.83) among migrant construction workers. Non-fatal injury rates were significantly higher among these migrants (850.4 vs. 63.79 per 10,000 workers, for migrants and non-migrants respectively in 2007) [28]. In Switzerland, Frickmann et al. [49] found that migrants sustained more injuries overall, although severe injuries (defined by Injury Severity Score greater than 15) were more frequent among Swiss workers (16% Swiss vs. 8% Non-Swiss). In Ireland, Connell et al. [50] reported that all eight cases of penetrating ocular trauma occurred in non-national workers due to injuries related to hammering, grinding, and splashing. Each case required surgery, with over half requiring multiple surgeries.

By contrast, three studies identified increased occupational injuries among non-migrant construction workers; two studies in Taiwan and Italy reported higher injury risk among non-migrant construction workers [29,64] and one study (United States) reported higher injury rates among non-migrants [37]. In Taiwan, for example, Wu et al. found that foreign construction workers had a 25% lower occupational accident rate than native workers (standard morbidity ratio 0.75; 95% CI 0.60-0.93) [29]. Similarly, Ricci et al.’s study in Italy observed that groups of migrants from Eastern Europe (OR 0.337; 95% CI 0.180-0.632) and Balkan countries (OR 0.508, 95% CI 0.259-0.998) had lower odds of reporting personal experience of occupational injury compared to native Italian construction workers [64]. Zhang et al. further found that native-born construction workers in the US had higher rates of non-fatal work injuries than foreign-born construction workers (non-migrant rate 214 per 10,000 workers, 95% CI 183–251 per 10,000 workers; migrant rate 104 per 10,000 workers, 95% CI 67–163 per 10,000 workers) [37].

Five studies (South Korea, United States, Italy) reported no significant differences between certain migrant and non-migrant groups [32,41,47,60,64].

Differences in data sources, sampled populations, and study periods may partially explain the variation in findings within and across countries. In Italy, for example, studies using national administrative registry data from the private sector (Giraudo et al. 2017, data sourced from 2000 to 2005; Giraudo et al. 2019, data sourced from 2005 and 2010) and a national labor force survey (Salvatore et al., data sourced from 2007) identified elevated injury risk among certain groups of migrants, while a survey of trainee workers at a single vocational school (Ricci et al., published in 2021, data collection period not reported) found lower odds of reported injury among Eastern European and Balkan migrants compared to local Italian workers [35,41,42,64]. Similarly, in the United States, a single-site study conducted at a metropolitan trauma hospital (Forst et al., qualitative interviews conducted in 2003) found higher counts of injuries among foreign-born workers, whereas a national household survey (Zhang et al., data sourced from 1997 to 2005) identified higher rates of injury among US-born workers [37,56]. In South Korea, a national compensation registry study (Cha and Cho, data sourced from 2005-2007) identified elevated non-fatal injury risk among migrants, while a survey of workers at selected construction sites conducted approximately 10 years later (Korkmaz and Park, data sourced from 2016) found no significant difference in injuries between foreign workers and Korean workers [28,60]. More information regarding study design and population is detailed in Table 1.

These contrasting findings are consistent with differences in ascertainment: registry- and survey-based studies capture formally reported injuries relative to workforce denominators, whereas hospital-based series and self-report samples reflect care seeking, reporting behavior, and workforce composition at a given site, each of which may differ between migrant and non-migrant workers; the site- and school-based surveys additionally relied on non-random samples. Temporal factors, including economic cycles and changes in migrant workforce composition and safety regulation, may further contribute to divergent results across study periods.

#### Fatal injuries.

Sixteen studies (South Korea, Israel, Australia, US, Thailand) reported occupational fatalities among migrant and non-migrant construction workers. Mortality outcomes included non-specific occupational fatalities (work-related deaths without a specified cause) (n = 11) [28,34,38–40,43,45–47,54,61], fatal falls (n = 3) [30,31,53], traumatic brain injury (TBI) (n = 1) [44], and heat-related fatalities (n = 1) [33] (see Tables 3 and 4).

**Table 3:** Rates of work-related fatal injury or illness among migrant and non-migrant construction workers.

Author, Year	Non-migrant Group	Migrant Group	Type of Injury	Non-Migrant Rate (95% CI)	Migrant Rate (95% CI)	Unit of Analysis (for Rate)
Cha, 2014	Korean	Migrant	Work-related fatal injury (2005)	2.56	40.75	per 10,000 workers
			Work-related fatal injury (2006)	2.2	15.16	per 10,000 workers
			Work-related fatal injury (2007)	1.94	46.33	per 10,000 workers
			Work-related fatal disease (2005)	0.4	2.55	per 10,000 workers
			Work-related fatal disease (2006)	0.37	1.52	per 10,000 workers
			Work-related fatal disease (2007)	0.32	8.97	per 10,000 workers
Dong, 2009	Hispanic, US-born	Hispanic, Foreign-born	Fatal falls	4.1	5.5	per 100,000 FTE workers
Dong, 2013	US-born	Foreign-born	Fatal falls from roof	0.99 (0.98, 0.99)	1.82 (1.79, 1.84)	per 100,000 FTE workers
Dong, 2019	US-born	Mexico-born	Heat-related death	0.13 (0.13, 0.14)	0.28 (0.27, 0.30)	per 100,000 FTE workers
		Born in “other” country			0.07 (0.07, 0.08)	per 100,000 FTE workers
King, 2024	Country of Origin: Australia	Country of origin: English-speaking countries	Suicide deaths^1^	25.9	19.6	per 100,000 workers
		Country of origin: Non-English speaking European countries			16	per 100,000 workers
		Country of origin: Other countries			15	per 100,000 workers
Konda, 2016	Hispanic, Native-born	Hispanic, Foreign-born	Fatal traumatic brain injury (TBIs)	2.7	3.8	per 100,000 FTE workers
	Non-Hispanic, Native-born	Non-Hispanic, Foreign-born		2.3	2.6	per 100,000 FTE workers
	Native-born (US)	Foreign-born		2.4	3.6	per 100,000 FTE workers
Steege, 2014	Not foreign-born	Foreign-born	Fatal Occupational Injuries	10.2	10.1	per 100,000 workers

^1^King, 2024: Age-standardized suicide rate (ASR).

**Table 4:** Prevalence of occupational morbidities and mortalities among migrant and non-migrant construction workers. *Values reported to two decimal places unless otherwise stated in original study.*

Author, Year	Sector	Non-migrant Group	Migrant Group	Type of Injury	Frequency of Injury (Native) [count/total (%)]	Frequency of Injury (Migrants) [count/total (%)]
Byler, 2013	Carpenter (construction trade worker)	Native-born Hispanic/Latino worker in US	Foreign-born Hispanic/Latino workers in US	Fatal injuries†	15/79 (18.99%)	64/79 (81.01%)
	Construction laborers (construction trade worker)				78/403 (19.35%)	325/403 (80.65%)
	Roofers (construction trade worker)				15/86 (17.44%)	71/86 (82.56%)
Byler, 2018	Construction and maintenance	US-born	Foreign-born	Occupational fatalities	8698/11012 (78.99%)	2314/11012 (21.01%)
Connell, 2007	Construction	Irish	Polish	Construction-related ocular injuries	80/155 (51.61%)	45/155 (29.03%)
			Romanian			5/155 (3.23%)
			Lithuanian			16/155 (10.32%)
			Other (non-Irish)			9/155 (5.81%)
Corvalan, 1994	Construction industry	Australian-born	Foreign-born from English-Speaking Countries	Work-related fatalities	111/182 (60.99%)	26/182 (14.29%)
			Foreign-born from Non-English-Speaking Countries			45/182 (24.73%)
Cruz, 2018	Construction industry	US-born	Foreign-born	Worker fatality, fatal falls	202/225 (89.78%)	23/225 (10.22%)
Davidson, 2009	Construction	Irish workers	Foreign-National workers	Work-related injuries	52/82 (63.41%)	30/82 (36.59%)
Dong, 2009	Construction	US-born	Foreign-born	Fatal falls†	1150/1641 (70.1%)	491/1641 (29.9%)
		Hispanic, US-born	Hispanic, Foreign-born		103/499 (20.6%)	396/499 (79 .4%)
Dong, 2013	Construction	US-born	Foreign-born	Fatal falls from roof†	582/875 (66.50%)	293/875 (33.50%)
				Other fatal falls†	1333/1844 (72.30%)	511/1844 (27.70%)
				All fatalities†	6255/8123 (77.00%)	1868/8123 (23.00%)
Dong, 2014	Construction	US-born	Foreign-born	Fatal falls†	2281/2986 (76.4%)	705/2986 (23.6%)
Dong, 2019	Construction	US-born	Mexico-born	Heat-related death	56/82 (68.29%)	21/82 (25.61%)
			Born in “other” country			5/82 (6.10%)
Forst, 2010	Construction	US-born	Foreign-born	Occupational injury	9/22 (40.91%)	13/22 (59.09%)
Frickmann, 2012	Construction	Swiss	Non-Swiss	Occupational accidents	263/782 (33.63%)	519/782 (66.37%)
Giraudo, 2017	Construction industry	Italians; i.e., HIC: individuals born in high-income countries	SMPC: individuals born in countries with strong migratory pressure (SMPC: Africa; Asia excluding Israel, South Korea and Japan; Latin America; Central and Eastern Europe)	Occupational injuries	1389/2457 (56.53%)	710/2457 (28.90%)
			of which born in Morocco			358/2457 (14.57%)
Giraudo, 2019	Construction industry	Italians; i.e., HIC: individuals born in high-income countries	SMPC: individuals born in countries with strong migratory pressure (SMPC: Africa; Asia excluding Israel, South Korea and Japan; Latin America; Central and Eastern Europe).	Occupational injuries (2005)	33/158 (20.89%)	125/158 (79.11%)
				Occupational injuries (2010)	49/152 (32.24%)	103/152 (67.76%)
Jeong, 2021	Shipbuilding	Korean-born	Foreigner	Occupational injury: death or disability	1944/2069 (93.96%)	125/2069 (6.04%)
Jung, 2023	Blue-collar construction workers	South Korean domestic	Foreign workers	Total injury claims	148404/158947 (93.4%)	10543/158947 (6.6%)
				Fatal injury claims	2905/3175 (91.5%)	270/3175 (8.5%)
				Non-fatal injury claims	145499/155772 (93.4%)	10273/155772 (6.6%)
				Fatal injury claims - male	2860/3124 (91.5%)	264/3124 (8.5%)
				Fatal injury claims - female	45/51 (88.2%)	6/51 (11.8%)
				Non-fatal injury claims - male	142184/152184 (93.4%)	10000/152184 (6.6%)
				Non-fatal injury claims - female	3315/3588 (92.4%)	273/3588 (7.6%)
King, 2024	Construction workers	Country of Origin: Australia	Country of origin: English-speaking countries	Suicide deaths	2618/3093 (84.64%)	251/3093 (8.12%)
			Country of origin: Non-English speaking European countries			103/3093 (3.33%)
			Country of origin: Other countries			121/3093 (3.91%)
Konda, 2016	Construction industry	Hispanic, Native-born	Hispanic, Foreign-born	Fatal traumatic brain injury (TBIs)	135/681 (19.82%)	546/681 (80.18%)
		Non-Hispanic, Native-born	Non-Hispanic, Foreign-born		1418/1522 (93.17%)	104/1522 (6.83%)
		Native-born (US)	Foreign-born		1560/2210 (71%)	650/2210 (29%)
Nuwayhid, 2003	Construction	Lebanese	Syrians	Work-related injuries (total)†	561/1623 (34.57%)	861/1623 (53.05%)
			Other Nationalities			201/1623 (12.38%)
		Lebanese	Syrians	Work-related injuries, hands†	138/405 (34.07%)	220/405 (54.32%)
			Other Nationalities			47/405 (11.60%)
		Lebanese	Syrians	Work-related injuries, feet†	82/289 (28.37%)	163/289 (56.40%)
			Other Nationalities			44/289 (15.22%)
		Lebanese	Syrians	Work-related injuries, eyes†	71/234 (30.34%)	132/234 (56.41%)
			Other Nationalities			31/234 (13.25%)
		Lebanese	Syrians	Work-related injuries, upper body†	138/352 (39.20%)	173/352 (49.15%)
			Other Nationalities			41/352 (11.65%)
		Lebanese	Syrians	Work-related injuries, lower body†	59/133 (44.36%)	63/133 (47.37%)
			Other Nationalities			11/133 (8.27%)
		Lebanese	Syrians	Work-related injuries, multiple parts†	23/69 (33.33%)	36/69 (52.17%)
			Other Nationalities			10/69 (14.49%)
		Lebanese	Syrians	Work-related injuries, information missing†	50/139 (35.97%)	72/139 (51.80%)
			Other Nationalities			17/139 (12.23%)
Pawthaisong, 2018	Construction	Thai workers	Foreign workers	Death (2016)1	93/105 (88.57%)	12/105 (11.43%)
				Disability (2016)1	48/52 (92.31%)	4/52 (7.69%)
				Minor Injury (2016)1	5424/6973 (77.78%)	1549/6973 (22.22%)
Salehi, 2022	Construction worker	Iranian	Non-Iranian	Electrical injuries/burns	49/66 (74.24%)	17/66 (25.76%)
Scrimshaw, 2003	Construction	Native-born Hispanic	Foreign-born Hispanic	Fatal Work Injuries†	397/1153 (34.43%)	756/1153 (65.57%)
	Construction laborers			Fatal Occupational Injuries†	137/490 (28%)	353/490 (72%)
	Roofers				31/105 (30%)	74/105 (70%)
	Carpenters, apprentices				25/92 (27%)	67/92 (73%)
Steege, 2014	Construction industry	Not foreign-born	Foreign-born	Fatal Occupational Injuries	4336/5674 (76.42%)	1338/5674 (23.58%)
Tuma, 2013	Construction industry	Qatar nationals	Non-nationals	Work-related falls†	8/281 (2.85%)	273/281 (97.15%)
Wu, 1997	Construction	Taiwanese	Foreign	Occupational injury	3358/3439 (97.64%)	81/3439 (2.36%)
		Taiwanese - male	Foreign - male		2774/2836 (97.81%)	62/2836 (2.19%)
		Taiwanese - female	Foreign - female		584/603 (96.85%)	19/603 (3.15%)
Yanai, 1999	Construction	Israeli	Palestinian	Fatal occupational accidents	38/101 (37.62%)	16/101 (15.84%)
			Foreign			47/101 (46.53%)
Zhang, 2009	Construction	US-born	Foreign-born	Work-related non-fatal injuries	174/197 (88.32%)	23/197 (11.68%)

†Prevalence data calculated by review authors based on data reported in included studies.

^1^Pawthaisong, 2018: National data regarding Death, Disability, and Minor Injury were extracted from the 2016 Thailand Workmen’s Compensation fund.

Among two studies reporting non-specific occupational fatalities, one identified higher fatality risk among migrant construction workers in South Korea (fatal injury crude risk ratio in 2007 23.89, 95% CI 16.63-34.33; fatal disease crude risk ratio in 2007 28.46, 95% CI 12.45-65.03), while another study identified no significant difference in the US (10.1 vs. 10.2 per 100,000 workers aged >15 years, for migrants and non-migrants respectively) [28,46]. Eight studies also described the distribution of occupational fatalities between migrant and non-migrant construction workers, with inconsistent findings [34,38–40,45,47,54,61].

Two US-based studies conducted by Dong et al. (2009; 2013) found that foreign-born construction workers had a higher risk of fatal falls from roofs (odds ratio [OR] 1.36, 95% CI 1.08-1.67) and a higher fatal fall risk index (1.56 vs 0.85 for native-born workers), where the risk index denotes the relative risk compared to the average risk of fatal falls from roofs for the construction industry [30,31]. TBIs represented an additional cause of fatal injury, with foreign-born construction workers experiencing a 50% higher rate of TBI (Rate Ratio [RR] 1.5, 95% CI 1.4-1.6) compared to their native-born counterparts [44]. Heat-related fatalities also disproportionately affected migrants, especially among those born in Mexico, who had more than twice the heat-related fatality risk of US-born workers (risk index 1.91 vs. 0.91), where the risk index represents relative risk compared to the average heat-related death risk for all construction workers between 2011–2016 [33].

### Other cause-specific injuries

Ocular injuries frequently affected construction workers. Nuwayhid et al. found that migrant workers in Lebanon experienced a higher proportion of eye injuries (15.4% of Syrians, 15.4% of Other nationalities, 12.7% of Lebanese) [36]. In Ireland, Connell et al. [50] reported comparable total counts of construction-related ocular injuries between Irish nationals (n = 80) and non-national workers (n = 75, comprised of 45 Polish, 5 Romanian, 16 Lithuanian, and 9 other workers), but greater severity of injuries among non-nationals. Specifically, all eight cases of penetrating ocular injury required surgical intervention and occurred in non-national workers, compared to none among Irish workers [50]. In contrast, Pawthaisong et al.’s study in Thailand observed a greater risk among non-migrant workers for eye splashes from miscellaneous objects or substances (Risk level 1.120 for Thai worker vs. 0.566 for foreign worker) [61]. Factors such as inadequate access, improper use, and limited enforcement of safety glasses contributed to the risk of eye injuries for both migrant and non-migrant workers [50,61]. In Ireland, Connell et al. reported variable use of safety glasses, with 41.2% of Irish nationals, 43% of Lithuanian migrants, and 0% of Romanian migrants wearing eye protection [50]. Migrants also faced increased vulnerability due to limited participation in safety training (90% of Irish nationals vs. 70% of non-nationals attending safety training), language barriers, and the absence of on-site interpreters [50].

Two studies examined the burden of musculoskeletal (MSK) injuries [52,63]. While there were no significant differences in the prevalence of MSK disorders between immigrant Turkish and native Dutch scaffolders in the Netherlands, Elders et al. cited back disorders (17% Dutch, 24% Turkish) and other musculoskeletal disorders (21% Dutch, 14% Turkish) as the primary cause for morbidity in both groups [52]. A 10-year follow-up study conducted in Germany conversely found that both general (all workers) and blue-collar (manual labor) migrant construction workers from Former Yugoslavia, Italy, Turkey, and other countries had lower rates of all-cause disability due to musculoskeletal disorders compared to native Germans (Standardized incidence ratios [SIRs] general workforce [GWF] migrants 1.34, 95% CI 1.06-1.67 vs. Germans 2.29, 95% CI 2.14-2.45; blue collar workforce [BCW] migrants 0.95, 95% CI 0.75-1.18 vs. Germans 1.62, 95% CI 1.52-1.73) [63].

Although all studies reported health outcomes by migrant status, only a subset described mechanisms of occupational injury, and even fewer disaggregated mechanisms by migrant status. Pawthaisong et al. reported that both foreign and native Thai workers were most at risk of occupational wounds and cuts involving sharp objects (Risk level 1.935 for Thai worker vs. 1.399 for foreign worker), while migrants were more at risk of injuries involving falling objects (Risk level 0.675 for Thai worker vs. 1.155 for foreign worker) [61]. In Lebanon, Nuwayhid et al. found that migrant workers from “Other nationalities” experienced significantly more wounds than Syrian and native Lebanese workers (37.3% of Other nationalities, 28.3% of Syrians, 20.5% of Lebanese, p < 0.001) [36]. Of note, electrical injuries and burns were identified as serious but understudied hazards for both migrant and non-migrant construction workers. A study by Salehi et al. found that native Iranian construction workers experienced more electrical injuries than non-Iranian construction workers (49/66 or 74.24% Iranian vs. 17/66 or 25.76% non-Iranian) [65].

### Morbidity

Mental health problems were an understudied occupational risk, with only three studies (Italy, Netherlands, Australia) directly comparing migrant and non-migrant construction workers’ mental health [48,52,58]. In Italy, migrant Ghanaian masons had significantly higher odds of interpersonal disorders compared to Italian workers (OR 3.134, 95% CI 1.507-6.518). Notably, Ghanaian mason workers were less likely to experience anxious-depressive disorders (OR 0.314, 95% CI 0.155-0.698) and poor general health outcomes (OR 0.095, 95% CI 0.046-0.197) compared to Italian workers [58].

In contrast, Elders et al. reported no differences in the prevalence of psychological disorders between immigrant Turkish (11/131, 8%) and native Dutch scaffolders (10/125, 8%) in the Netherlands [52]. In Australia, King et al. found that native construction workers had higher suicide rates (age-standardized suicide rate [ASR] 25.9 per 100,000 workers) compared to foreign nationals born in English-speaking countries (ASR 19.6 per 100,000 workers) and non-English speaking countries (ASR 16.0 per 100,000 workers) [48].

Four studies examined cardiovascular and metabolic diseases [52,57,59,62]. In the Netherlands, Elders et al. reported that immigrant Turkish scaffolders (11/131, 8%) had more cardiovascular diagnoses compared to native Dutch workers (5/125, 4%), potentially explained by age and employment duration [52]. Chung et al.’s study in Hong Kong found no significant differences between minority migrants or mainland Chinese workers and Hong Kong–born workers [59]. By contrast, Arndt et al.’s (2007) study in Germany observed variable outcomes among migrants from different ethnic groups. For example, construction workers from the former Yugoslavia had higher odds of being overweight (OR 1.246, 95% CI 1.122-1.384), while migrant workers categorized as “Other” had lower odds compared to German workers (OR 0.747, 95% CI 0.638-0.876) [57]. In contrast, no significant differences in BMI were observed between Italian migrant or Turkish migrant construction workers and the local German workers [57]. Similarly, no significant differences in circulatory disease-related mortality were found between native Germans and migrants in the total cohort [62].

All-cause morbidity, mortality, and long-term health outcomes were infrequently examined [52,62,63]. Two German cohort studies by Arndt et al. (2004, 2005) found significantly lower all-cause morbidity (SIR GWF migrant 1.04, 95% CI 0.91-1.19; SIR GWF non-migrant 1.54, 95% CI 1.48-1.61) and mortality (Standardized mortality ratio [SMR] migrant 0.57, 95% CI 0.49-0.67; SMR non-migrant 0.75, 95% CI 0.70-0.81) among migrant workers [62,63]. Elders et al. further noted that pulmonary diseases were diagnosed more in Turkish workers (n = 8 Turkish vs. n = 3 Dutch scaffolders) and malignancies were only diagnosed in Dutch workers (4 Dutch workers) [52].

All above data regarding prevalence and risk of occupational morbidity and mortality among migrant and non-migrant construction workers is listed in Table 4 and Table 5.

**Table 5:** Risk of occupational morbidities and mortalities among migrant and non-migrant construction workers. *Respective measure of effect corresponds to type of worker and type of injury in row, unless otherwise stated.*

Author, Year	Sector	Non-migrant Group	Migrant Group	Type of Injury	Measure of Effect				
					OR (95% CI, p value)	Type of Misc Measure of Effect (1)	Value (95%CI)	Type of Misc Measure of Effect (2)	Value (2) (95%CI)
Arndt, 2004	Construction (plumbers, carpenters, painters, plasterers, bricklayers, and unskilled workers/labourers)	German	Former Yugoslavia, Italian, Turkish, Other	All deaths	–	Standardized mortality ratio (SMR) - German	0.75 (0.70, 0.81)	Standardized mortality ratio (SMR) - Former Yugoslavia, Italian, Turkish, Other	0.57 (0.49, 0.67)
				Cancer	–		0.96 (0.85, 1.09)		0.64 (0.46, 0.87)
				Circulatory systems	–		0.62 (0.53, 0.73)		0.50 (0.34, 0.72)
				Respiratory system	–		0.61 (0.37, 0.96)		0.59 (0.16, 1.51)
				Digestive system	–		0.75 (0.56, 0.98)		0.55 (0.27, 1.02)
				Injury and poisoning	–		0.94 (0.74, 1.19)		0.81 (0.44, 1.30)
				All non-transport accidents	–		1.60 (1.06, 2.31)		1.79 (0.77, 3.53)
				Falls	–		2.04 (1.21, 3.23)		1.32 (0.27, 3.86)
				Struck by falling objects	–		1.35 (0.44, 3.15)		4.28 (1.17, 11.01)
Arndt, 2005	Construction: general work force (GWF)	German	Former Yugoslavia, Italian, Turkish, Other	All-cause disability	–	Standardized incidence ratio (SIR) GWF - German	1.54 (1.48, 1.61)	Standardized incidence ratio (SIR) GWF -Former Yugoslavia, Italian, Turkish, Other	1.04 (0.91, 1.19)
	Construction: blue collar workers (BCW)				–	Standardized incidence ratio (SIR) BCW - German	1.17 (1.12, 1.22)	Standardized incidence ratio (SIR) BCW - Former Yugoslavia, Italian, Turkish, Other	0.78 (0.69, 0.89)
	Construction: general work force (GWF)			Disability due to musculoskeletal disorders	–	Standardized incidence ratio (SIR) GWF - German	2.29 (2.14, 2.45)	Standardized incidence ratio (SIR) GWF - Former Yugoslavia, Italian, Turkish, Other	1.34 (1.06, 1.67)
	Construction: blue collar workers (BCW)				–	Standardized incidence ratio (SIR) BCW - German	1.62 (1.52, 1.73)	Standardized incidence ratio (SIR) BCW - Former Yugoslavia, Italian, Turkish, Other	0.95 (0.75, 1.18)
	Construction: general work force (GWF)			Disability due to accidents	–	Standardized incidence ratio (SIR) GWF - German	2.71 (2.18, 3.33)	Standardized incidence ratio (SIR) GWF - Former Yugoslavia, Italian, Turkish, Other	1.31 (0.57, 2.58)
	Construction: blue collar workers (BCW)				–	Standardized incidence ratio (SIR) BCW - German	1.97 (1.59, 2.42)	Standardized incidence ratio (SIR) BCW - Former Yugoslavia, Italian, Turkish, Other	0.95 (0.41, 1.88)
Arndt, 2007	Construction	German	Former Yugoslavian	BMI (Ideal -BMI < 24.9 kg/m2) = outcome NO, Intermediate/Poor BMI >=25 kg/m2) = outcome YES)	1.246 (1.122, 1.384)†	–	–	–	–
			Italian		0.975 (0.868, 1.095)†	–	–	–	–
			Turkish		0.992 (0.879, 1.120)†	–	–	–	–
			Other		0.747 (0.638, 0.876)†	–	–	–	–
Capasso, 2018	Masons	Italian	Ghanaian	Interpersonal disorders	3.134 (1.507, 6.518) *p < 0.05.	–	–	–	–
				Anxious-depressive disorders	0.314 (0.155, 0.698) *p < 0.05.	–	–	–	–
				General health	0.095 (0.046, 0.197) *p < 0.05.	–	–	–	–
Cha, 2014	Construction	Korean	Migrant	Work-related fatal injury (2005)	15.97 (11.173, 22.826)†	Crude risk ratio	15.91 (11.13, 22.74)	–	–
				Work-related fatal injury (2006)	6.89 (4.774, 9.957)†		6.89 (4.77, 9.94)	–	–
				Work-related fatal injury (2007)	24 (16.701, 34.480)†		23.89 (16.63, 34.33)	–	–
				Work-related non-fatal injury (2005)	4.89 (4.339, 5.520)†		4.76 (4.22, 5.36)	–	–
				Work-related non-fatal injury (2006)	2.83 (2.551, 3.131)†		2.79 (2.52, 3.09)	–	–
				Work-related non-fatal injury (2007)	14.48 (13.270, 15.796)†		13.33 (12.22, 14.54)	–	–
				Work-related fatal disease (2005)	6.38 (1.569, 25.918)†		6.38 (1.57, 25.91)	–	–
				Work-related fatal disease (2006)	4.15 (1.316, 13.117)†		4.15 (1.32, 13.12)	–	–
				Work-related fatal disease (2007)	28.48 (12.462, 65.083)†		28.46 (12.45, 65.03)	–	–
				Work-related non-fatal disease (2005)	5.53 (2.465, 12.420)†		5.53 (2.46, 12.41)	–	–
				Work-related non-fatal disease (2006)	1.96 (1.080, 3.555)†		1.96 (1.08, 3.56)	–	–
				Work-related non-fatal disease (2007)	8.06 (5.054, 12.857)†		8.04 (5.04, 12.83)	–	–
Chung, 2018	Construction	Hong Kong-Born Chinese	Mainland-born Chinese	Weight Status [Intermediate/Poor (BMI>=23 kg/m2]	0.821 (0.505, 1.332)†	Cardiovascular Health Score - Chinese— Hong-Kong Born in Hong Kong or long-term resident	3.06 (sd: 1.15, p value = 0.55)	Cardiovascular Health Score - Chinese - Mainland	3.19 (sd: 1.12, p value = 0.55)
			Minority (from Nepal, Pakistan, or other countries)		2.092 (0.760, 5.760)†				
			Mainland-born Chinese	Blood Cholesterol [Intermediate/Poor (>=5.2 mmol/L or 200 mg/dL)]	0.591 (0.339, 1.033)†				
			Minority		0.538 (0.197, 1.474)†				
			Mainland-born Chinese	Blood Pressure [Intermediate/Poor (>=120mmHg/ 80mmHg)]	0.725 (0.396, 1.326)†			Cardiovascular Health Score - Minority	2.96 (sd: 0.88, p value = 0.55)
			Minority		1.673 (0.376, 7.440)†				
			Mainland-born Chinese	Blood Glucose [Intermediate/Poor (>=5.6 mmol/L or 100 mg/dL)]	0.586 (0.205, 1.677)†				
			Minority		0				
Dong, 2009	Construction	Hispanic, US-born	Hispanic, Foreign-born	Fatal falls	1.36 (1.08, 1.67)	Adjusted OR^1^	1.31 (1.05, 1.62)	–	–
Dong, 2013	Construction	Native (US-born)	Foreign-born	Fatal falls from roof	–	Risk index - native^2^	0.85	Risk index - foreign-born^2^	1.56
Dong, 2015	Blue-collar construction occupations	US-born	Foreign-born	Worker injury	1.124 (0.639, 1.977)†, p-value = 0.772	–	–	–	–
				Injury resulting in no days away from work	1.349 (0.607, 2.996)†, p-value = 0.772	–	–	–	–
				Injury resulting in days away from work	1.063 (0.568, 1.989)†, p-value = 0.772	–	–	–	–
Dong, 2019	Construction	US-born	Mexico-born	Heat-related death	–	US Born Risk Index^3^	0.91, α > 0.05	Mexican Born Risk Index^3^	1.91, α < 0.05
			Born in “other” country		–			Other Country Born Risk Index^3^	0.52, α > 0.05
Elders, 2004	Scaffolds	Dutch	Turkish	Back disorders	1.60 (0.865, 2.962)†	Age-adjusted RR for disability^4^	2.48 (1.94, 3.18)	Age-adjusted RR for disability (survival analysis)	2.45 (1.12, 5.35)
				Other musculoskeletal disorders	0.61 (0.314, 1.172)†				
				Accidents	0.88 (0.416, 1.867)†				
				Psychiatric disease	1.05 (0.431, 2.577)†				
				Cardiovascular disease	2.20 (0.742, 6.524)†				
				Other disorders	1.01 (0.502, 2.025)†				
				Unknown disorders	0.78 (0.428, 1.425)†				
Giraudo, 2017	Construction	Italians; i.e., HIC: individuals born in high-income countries	SMPC: individuals born in countries with strong migratory pressure (SMPC: Africa; Asia excluding Israel, South Korea and Japan; Latin America; Central and Eastern Europe).	Occupational injuries	–	Incidence rate ratio HIC^5^	1	Incident rate ratio SMPC^5^	1.10 (0.98, 1.23)
			of which born in Morocco		–			Incident rate ratio Morocco^5^	1.41 (1.24,1.60)
Giraudo, 2019	Construction	Italians; i.e., HIC: individuals born in high-income countries	SMPC: individuals born in countries with strong migratory pressure (SMPC: Africa; Asia excluding Israel, South Korea and Japan; Latin America; Central and Eastern Europe).	Occupational injuries (2005)	–	Incident rate ratio HIC 2005^6^	1.19 (0.94, 1.50)	Incident Rate Ratio SMPC 2005	1
				Occupational injuries (2010)	–	Incident rate ratio HIC 2010	0.22 (0.15, 0.33)	Incident Rate Ratio SMPC 2010	0.84 (0.65, 1.09)
Jeong, 2021	Shipbuilding	Korean-born	Foreigner	Occupational injury: death or disability	1.685 (1.116, 2.544), p-value = 0.013	–	–	–	–
King, 2024	Construction	Country of Origin: Australia	Country of origin: English-speaking countries	Suicide death	–	Incidence rate ratio (IRR) Australian origin	1.76 (1.68, 1.84)	Incidence rate ratio (IRR) - English-speaking countries origin	1.64 (1.41, 1.89)
			Country of origin: Non-English speaking European countries		–			Incidence rate ratio (IRR) - Non-English-speaking countries origin	1.43 (1.14, 1.8)
			Country of origin: Other countries		–			Incidence rate ratio (IRR) - Other countries origin	2.22 (1.81, 2.7)
Konda, 2016	Construction industry	Hispanic, Native-born	Hispanic, Foreign-born	Fatal traumatic brain injury (TBIs)	–	Rate ratio	1.4 (1.2, 1.7)	–	–
		Non-Hispanic, Native-born	Non-Hispanic, Foreign-born		–		1.1 (0.9, 1.3)	–	–
		Native-born (US)	Foreign-born		–		1.5 (1.4, 1.6)	–	–
Korkmaz, 2018	Construction	Korean domestic workers	Foreign workers	Workplace accidents	0.802 (0.434, 1.482)†	–	–	–	–
Ricci, 2021	Construction	Italy (Region of Origin)	North Africa (Algeria, Egypt, Morocco, Tunisia)	Previous personal experience of occupational injury	0.434 (0.142, 1.323)†	–	–	–	–
			Eastern Europe (Lithuania, Poland, Moldova, and Romania)		0.337 (0.180, 0.632)†	–	–	–	–
			Balkans (Albania, Croatia, Kosovo, Serbia)		0.508 (0.259, 0.998)†	–	–	–	–
Salvatore, 2012	Construction (skilled and unskilled construction work)	Italians	Immigrants	Work-related injuries	2.05 (1.56, 2.69)	–	–	–	–
	Unskilled construction work				8.64 (2.85, 26.2)	–	–	–	–
Wu, 1997	Construction	Taiwanese	Foreign	Occupational injury	–	Standard morbidity ratio (SMR)^7^	0.75 (0.60, 0.93)	–	–
		Taiwanese - male	Foreign - male	Occupational injury	–		0.41 (0.32, 0.51)	–	–
		Taiwanese - female	Foreign - female	Occupational injury	–		380 (243.3, 593.6)	–	–

† OR and 95% CI calculated by review authors based on data reported in included studies. Calculated OR and 95% CI reported with 3 decimals for precision.

1 Dong, 2009: OR for foreign-born status from a multiple logistic regression adjusting for ethnicity, age, job tenure, establishment size, employment type, project type, occupation, and region; the unadjusted OR (1.36) compares foreign-born with US-born Hispanic workers.

^2^Dong, 2013: “The average risk of fatal falls from roofs for the construction industry was used as the reference (risk = 1).^”^

^3^Dong, 2019: “The average risk of heat‐related deaths (among all construction workers) between 2011 and 2016 was used as the reference (risk index = 1).”

^4^Elders, 2004: Relative risks estimated over 1981–2000 by Poisson regression and over 1997–2000 by survival analysis.

^5^Giraudo, 2017: IRR for HIC = 1. IRR adjusted for “age, firm geographic area, firm size (yearly average number of employees), and years of work.”

^6^Giraudo, 2019: Incidence rate ratios are relative to SMPC construction workers in 2005 (reference group). In 2010, the IRR comparing SMPC with HIC workers was 3.83 (95% CI 2.52-5.75).

^7^Wu, 1997: SMR “adjusted based on age distribution” and “calculated based on Poisson distribution.”

### Demographic factors associated with occupational health outcomes

Four US studies controlled for ethnicity and race in their analyses. There was strong evidence that foreign-born Hispanic construction workers disproportionately experienced occupational fatalities compared to native-born Hispanic workers [31,38,44,45]. Dong et al. (2009) found that foreign-born status (OR 1.31, 95% CI 1.05-1.62, p 0.015) was a stronger predictor of fall-related mortality than ethnicity (OR 1.04, 95% CI 0.84-1.28, p 0.724) [31]. Konda et al. also highlighted the ongoing risks faced by Hispanic construction workers, reporting that Hispanic foreign-born workers faced a 40% higher rate of TBIs (rate ratio [RR] 1.4, 95% CI 1.2-1.7), with no significant differences between non-Hispanic native and foreign-born workers (RR 1.1, 95% CI 0.9-1.3) [44].

Several studies stratified findings by migrant country of origin, with morbidity and mortality outcomes widely varying across nationalities and health outcomes [34,36,41,48,50,54,57,59,64].

Age was also a determinant of occupational injuries among migrant and non-migrant construction workers [52,60]. One study in Korea by Korkmaz and Park [60] reported older foreign construction workers experienced significantly fewer accidents (t -2.016, p < 0.05), while age was not significantly associated with accident risk among native Korean workers (t -0.589). Older foreign workers were more likely to perceive risk controls as obstructive to their work responsibilities (Pearson’s correlation coefficient (r) -0.330, p < 0.01). This association was not significant among Korean workers (r 0.054), suggesting age-related differences in attitudes toward safety measures by nativity [60].

The role of gender in shaping occupational risks among migrant and non-migrant construction workers remains underexplored, as study samples were all or predominantly male (n = 36). Wu et al. specifically found that female migrant construction workers in Taiwan had a higher injury risk (Standard Morbidity Ratio 380, 95% CI 243.3-593.6) compared to male migrants (Standard Morbidity Ratio 0.41, 95% CI 0.32-0.51), relative to their native-born counterparts [29].

### Other factors associated with adverse occupational health outcomes

Three studies (Australia, South Korea) linked limited language proficiency to adverse occupational health outcomes [34,48,60]. In Australia, Corvalan et al. found that migrant workers from non-English speaking countries faced higher occupational fatality risk during the first five years of residence, with the construction sector accounting for 24.7% of deaths in this group, compared to 17.2% among English-speaking migrants and 12.6% among Australian-born workers [34]. Korkmaz and Park further identified poor language proficiency as a strong predictor of accident risk for foreign workers in South Korea (t -5.08, p < 0.001) [60]. King et al.’s study in Australia conversely reported that suicide mortality rates were higher among Australian-born construction workers (ASR = 25.9 per 100,000) compared to migrant workers between 2006 and 2020 [48]. Among migrants, individuals from English-speaking countries (19.6 per 100,000) had higher rates of suicide than those born in non-English speaking countries (16.0 per 100,000) [48]. However, data from 2006 to 2020 showed that construction workers born in Australia and English-speaking countries had more stable suicide rates over time, while those born in non-English speaking countries or other countries showed more variability, with rates converging toward native and English-speaking country rates [48]. Twenty-five studies from our review speculated that language barriers played a role in adverse health outcomes among migrant construction workers but did not report data to support these hypotheses [28–31,35–42,44,45,47,49–52,54–56,58,61,64].

Psychosocial and cultural factors also influenced observed health disparities. In Italy, Capasso et al. [58] described associations indicating that Ghanaian masons who reported racial discrimination or higher perceived work demands were more likely to report interpersonal disorders than Italian masons. While the study did not provide quantitative measures of these effects, the authors noted that Ghanaian masons who maintained stronger ties to their culture of origin reported fewer anxiety-related symptoms; however, they remained more prone to interpersonal disorders compared to Italian masons [58]. Ricci et al.’s study investigated how ethnic background and cultural orientation shaped perceptions of workplace safety and injury risk in Italy [64]. North African construction workers reported greater job-related pressure and insecurity (OR 1.752, p 0.040) but had no significant differences in baseline injury, defined by previous injury experience (OR 0.434, 95% CI 0.142-1.323) compared to native Italian construction workers. Eastern European (OR 0.337, 95% CI 0.180-0.632) and Balkan (OR 0.508, 95% CI 0.259-0.998) construction workers reported lower rates of injury compared to Italian workers, along with reduced awareness of workplace hazards among Eastern Europeans (OR 0.635, p 0.002) and more risk-taking attitudes among both Eastern Europeans and Balkans (Eastern Europe OR 1.441, p 0.011; Balkan OR 1.601, p 0.007); only Balkan construction workers perceived the safety conditions more positively than Italian construction workers (OR 1.611, p 0.005) [64].

Several employee level characteristics, such as years of employment and work experience, also influenced occupational health risks. In Italy, Salvatore et al. found that male immigrant construction workers faced the highest injury risk during the first year (OR 3.67, 95% CI 1.44-9.34), lower risks mid-career (OR 1.28, 95% CI 0.71-2.32), and an elevated risk again after 10 or more years (OR 4.84, 95% CI 1.65-14.15), compared to native Italian workers who experienced a stable injury rate across their period of employment [35]. Giraudo et al. (2017) found that injury rates among native Italian construction workers declined with increasing job tenure, while rates among migrants born in countries with strong migratory pressure and Moroccan workers declined initially (at 6–12 months), then increased and remained elevated after 13 months of employment [41]. In South Korea, the study by Jeong indicated that foreign workers in the shipbuilding industry had disproportionately higher odds of accidents leading to death or disability (OR 1.685, p 0.013), particularly those with less than one year of experience (105/125 or 84% of Foreigners injured vs 1194/1944 or 61.4% of Koreans injured) [43].

Four studies indicated that employee and employer level characteristics, such as type of construction work, access to and comprehension of safety training, and use of protective gear, exacerbated workers’ health risks [35,50,60,61]. In Italy, Salvatore et al. found the odds of injury were twice as high for immigrant construction workers overall (OR 2.05, 95% CI 1.56-2.69) and nearly nine times higher for unskilled immigrant laborers (OR 8.64, 95% CI 2.85-26.20) compared to native workers [35]. In South Korea, Korkmaz and Park found that less training was associated with increased accidents among native Korean construction workers (t -4.499, p < 0.001) but not foreign workers (t 0.048) [60]. The same study found that attendance of safety briefings was a stronger predictor of protective gear use among foreign construction workers (r 0.273, p < 0.01) compared to native workers (r 0.132, p < 0.01) [60]. Moreover, the study reported that lower educational attainment was associated with poorer safety discipline (r -0.198, p < 0.05) and higher accident rates (t -6.488, p < 0.001) among foreign construction workers [60].

## Discussion

The purpose of our review was to synthesize global evidence on occupational health disparities between migrant and non-migrant construction workers. Construction is consistently recognized as one of the most hazardous occupations worldwide, often reporting higher rates of injury and fatality compared to other labor-intensive industries, like manufacturing [1]. This review shows that migrants experience a disproportionate burden of associated morbidity and mortality, consistent with the broader global evidence on migrant worker occupational health [22,66].

Furthermore, our review highlights that migrant construction workers experience higher rates of fatal injury and work-related illness, including fatal falls, TBI, heat-related fatalities, and ocular injuries, across middle and high-income countries. Some studies reported lower all-cause morbidity and mortality among migrant construction workers [62,63]. There were inconsistent findings in regard to non-fatal injuries and mental health problems, with some studies identifying greater rates among migrant construction workers and others among non-migrants, which may reflect variable access to care and underreporting rather than true health outcomes or needs. The mixed evidence demonstrates that all workers are exposed to hazardous working conditions in the construction sector; there is a need to improve occupational health and safety universally for all workers. However, the disparities between migrant and non-migrant workers, especially for fatal outcomes, suggest that migrant status constitutes a significant structural determinant of occupational health in physically demanding industries.

Our findings indicate that migrant construction workers’ health disparities stem primarily from structural factors embedded in the construction labor market. Migrants are disproportionately employed in small firms, informal or subcontracted arrangements, and newly hired or temporary positions. These contexts are often marked by weak regulatory oversight, inconsistent safety enforcement, and inadequate access to protective equipment, fall-prevention systems, and formal safety training. These systemic deficiencies reflect broader inequities in labor protections and heighten the risk of migrants’ occupational injury and death.

Social and cultural barriers, including limited language proficiency, cultural dislocation, and experiences of racial discrimination, further compound these risks by reducing workers’ ability to understand safety protocols, assert their rights, or seek redress. For example, even when safety training is provided, it is often poorly adapted to migrants’ language, education, or literacy levels, limiting comprehension and practical uptake. When safety information and supervision are not adapted for multilingual or culturally diverse workforces, it can translate directly into higher injury rates and psychological strain [67].

Individual factors, including age, gender, and work experience, can exacerbate these structural constraints. Younger workers may face elevated injury risk due to inexperience and lack of training, whereas older migrant workers accumulate physical strain over time and may face declining access to physically less demanding roles [31]. Migrant women in construction often face unique vulnerabilities linked to unstable employment contracts, gendered occupational expectations, and limited access to protective gear or gender-appropriate sanitation facilities. These conditions put migrant women at greater risk of injury, disease, fatigue, and psychological strain [9,68,69]. Migrant workers with limited construction experience are also more likely to perform hazardous tasks without adequate supervision, increasing their susceptibility to occupational accidents and injury [69]. Together, these intersecting structural and individual factors reinforce an unequal risk environment in the construction sector, in which migrant status itself operates as a key determinant of occupational health.

### Limitations

This review presents several limitations. Studies conducted in low-income countries and those published in foreign languages were excluded at the screening stage due to insufficient focus on the construction sector and limited comparison between migrant and non-migrant construction workers. The lack of non-English literature or studies conducted in low-income countries reduces the global applicability of our findings, given the large, often undocumented, scale of labor migration in lower-income countries [70]. The majority of included studies were conducted in high-income countries (n = 34), with three studies from upper-middle-income settings (Iran, China, Thailand) and only one from a lower-middle-income country (Lebanon), precluding meaningful comparisons across income settings. Combined with the lack of disaggregated data, this limits understanding of health outcomes among the most marginalized migrant worker groups, including gender minorities, refugees, asylum seekers, and undocumented migrants.

Definitions of ‘migrant status’ and ‘construction work’ varied considerably across included studies. Some studies classified migrants by ‘place of birth’, others by ‘citizenship' and one included internal migrants. Several papers grouped construction work and extraction occupations together. Methodological inconsistencies limited comparability and may have introduced heterogeneity across reported outcomes.

The available evidence may underestimate the true morbidity and mortality burden among migrant construction workers, due to reporting bias and underreporting linked to limited health care access, lack of legal protections, language barriers, and fear of deportation. Further, many studies analyzed data from administrative registries and health insurance or disability compensation claims, introducing selection bias through probable exclusion of certain migrant subgroups, such as temporary and undocumented migrants. Another consideration is the salmon bias effect, whereby migrants experiencing adverse health return to their country of origin [71]. Importantly, these factors are likely to vary across and within countries, owing to variation in immigration policies, pathways to legal status, and access and availability of public health care and social protection. Together, these factors might explain the variation in occupational morbidity and mortality, particularly non-fatal injury risk, observed.

Finally, variation in study designs, exposure and outcome definitions, and effect measures limited comparability across studies and precluded meta-analytic estimation of the additional risk associated with migrant status. Given the limited evidence base, we adopted broad inclusion criteria and did not exclude studies on the basis of appraised quality; as a result, some lower-quality studies were included. In addition, many studies relied on non-probability sampling (e.g., purposive or convenience samples) and had small sample sizes, particularly those conducted in hospital and construction-site settings, limiting statistical power and generalizability; the larger registry- and cohort-based studies were in turn constrained by the limitations of administrative data. Many studies also lacked adjustment for key confounders including job tenure, firm size, socioeconomic status, language proficiency, undocumented status, type of construction work, and length of time in host country, reducing the robustness of reported effect estimates and limiting the attribution of observed disparities specifically to migrant status. Collectively, these limitations underscore the need for rigorous research directly comparing outcomes between migrant and non-migrant construction workers to inform future resource allocation and policy.

### Implications for policy

Our findings confirm that the incidence and severity of work-related injuries and illnesses among migrants are strongly tied to structural forces rather than individual risk factors. Clinicians, employers, and policy makers must consider the compounded risks that arise at the intersection of migrant status and construction work [24]. In fact, practitioners must consider that many migrant construction workers are men and adolescent boys who are deceptively recruited or coerced into exploitative labor conditions that meet international definitions of labor trafficking [72]. Upstream interventions aligned with Sustainable Development Goal 8.8, which calls for “safe working environments and the protection of labor rights,” are urgently needed [24,73].

At the government and civil society level, major reforms are needed to address gaps in implementation and enforcement of labor safety regulations. Existing data and surveillance systems remain inadequate for capturing the full scale of migrant construction labor, partly because migration seldom occurs linearly or permanently, complicating efforts to classify migrants into well-defined subgroups [74,75]. Regional labor migration in low- and middle-income countries is particularly underrepresented in official statistics, owing to regional labor and trade agreements, in addition to undocumented migration [15]. Without reliable, disaggregated data, governments cannot accurately assess occupational risks and allocate resources that reflect the true burden of migrant labor. Surveillance systems should be restructured to disaggregate data by migrant status, visa type, employment setting (formal vs. informal), and demographics, while ensuring such data is not misused for immigration enforcement purposes.

Weak enforcement of labor protections and coercive recruitment systems underscore the need for coordinated labor inspection and anti-trafficking oversight. In the US, for example, limited audits of protective gear use and insufficient enforcement of Occupational Safety and Health Administration (OSHA) regulations in small residential construction reveal gaps in national labor protections [53]. Legislation should be revised to ensure that occupational injury reporting systems are accessible to migrant and undocumented workers [28,30,36,37,41,42,45,56,76–79]. Expanding access to affordable virtual translation services and legal support may reduce confusion and fear of deportation, and empower workers to report both non-fatal and fatal occupational health concerns or labor rights violations without retaliation from their employers [15,45,77]. However, both of these measures are unlikely to entirely alleviate these concerns unless there are broader structural reforms that decouple labor protections from immigration status.

Policy and labor reforms for migrant construction workers are colliding with increasingly hostile political climates. Anti-immigrant sentiment is exacerbated by certain labor policies, such as E-Verify mandates in several US states [80], and by health policies, such as UK regulations requiring healthcare workers to report patients’ immigration status, undermining principles of ethical practice [81,82]. These policies can deter migrants from reporting unsafe or exploitative conditions and accessing healthcare, undermining both individual and public health [77,78]. These problems are further compounded by financial pressures, as many low-wage migrants are exploited to pay substantial recruitment and visa fees [83]. In Gulf Cooperation Council countries, visa-linked sponsorship systems, such as the kafala model [84], grant employers unilateral control over the workers’ legal status, enabling exploitation and limiting worker rights. Such policies are counterintuitive amid increasing global reliance on migrant construction labor [10] but persist because global economic expansion and border politics outweigh labor and migrant rights.

Employers, particularly in small and residential firms, also have a direct role to play in improving migrants’ occupational health and safety. Research suggests that to minimize occupational hazards while addressing language barriers, firms should implement interventions that incorporate multilingual and visually based training sessions, access to low-cost translators, and cultural sensitivity in safety communication [13,85]. These workplace interventions can help alleviate stressors contributing to migrant construction workers’ poor mental health, but must be accompanied by employer training on mental health awareness and programs that facilitate workers’ access to routine health screenings and psychosocial support [86].

### Future research

Governments, funders, and research institutes must expand the range of occupational health risks that are monitored, analyzed, and prioritized for investment, moving beyond physical injuries to include psychological harms and outcomes and emerging environmental risks. Notably, only one study in this review examined heat-related illness [33], despite growing evidence that exposure to extreme temperatures poses serious health threats to construction workers. These risks are exacerbated by long working hours, insufficient rest breaks, and limited access to shade and hydration. Climate change compounds them further by intensifying the frequency and severity of extreme weather globally [87]. Evidence from the Qatar FIFA World Cup further demonstrates increased risk of heat stress and cardiovascular mortality among migrant construction workers, linked to heat exposure [88,89]. These risks are projected to intensify with climate change and stand to affect the construction sector and migrant workers substantially, as noted in a joint report by the World Health Organization and World Meteorological Organization on climate change and worker health [90].

Future studies should also prioritize structural and social health factors affecting migrant workers’ occupational health outcomes, including visa type, duration of residency, union membership, employment contracts, job tenure, and social integration, which likely influence exposure and access to social and legal protections. Analyses directly comparing outcomes between migrant and non-migrant workers, disaggregated by demographic characteristics like gender, country of birth, ethnicity, and mechanisms of injury, will additionally enable rigorous meta-analytic estimates and demographically responsive analysis and policy development. Ultimately, high-quality interventional research, including pragmatic trials and quasi-experimental studies, is urgently needed to identify low-cost, scalable solutions for reducing occupational morbidity and mortality among migrant construction workers.

Finally, more research is needed in low- and lower-middle-income countries, particularly to capture more marginalized groups, such as refugees and forcibly displaced people, especially in the context of ongoing conflicts and climate-driven migration. While a small number of included studies noted workers as young as sixteen or seventeen years old in their sample, no study examined child construction workers as a distinct population or reported outcomes disaggregated for migrant and non-migrant construction workers under 18. Several studies also did not report participant age, meaning some samples may have included minors. Children engaged in construction work therefore remain an understudied and potentially highly vulnerable group, and future studies should report participant age and examine outcomes for workers under 18. Collecting disaggregated data by age, sex, and migration status, in line with recommendations from the Global Compact for Safe, Orderly and Regular Migration, is essential for illuminating the unique risks and outcomes experienced by different migrant subgroups and developing tailored policy and interventions [76].

## Supporting information

S1 File PRISMA checklist. (DOCX)

S1 Table Quality appraisal. Adapted Joanna Briggs Institute (JBI) quality appraisal scoring criteria and risk of bias data for included studies.
(XLSX)

S1 Text Search terms. Example search strategy used in the review.
(DOCX)

S2 Text Definitions of key search terms in inclusion criteria. Operational definitions of key terms used in the search strategy and inclusion criteria.
(DOCX)

S3 Text Study selection. Detailed inclusion and exclusion criteria for study selection.
(DOCX)

## References

[1] International Labour Organization. Safety and health in construction: ILO code of practice, revised edition. Geneva: International Labour Organization; 2022.

[2] Dodoo JE, Al-Samarraie H. A systematic review of factors leading to occupational injuries and fatalities. J Public Health (Berl). 2023;31(1):99–113. doi: 10.1007/s10389-020-01427-4

[3] Syron L, Teske T, Kloczko D, Check P, Flynn MA, Sadeghpour N, et al. Safety and health at work: Construction workers. Cincinnati, OH: U.S. Department of Health and Human Services, Centers for Disease Control and Prevention, National Institute for Occupational Safety and Health; 2023. DHHS (NIOSH) Publication No. 2023-115. 10.26616/NIOSHPUB2023115

[4] Onarheim KH, Phua KH, Babar ZR, Flouris AD, Hargreaves S. Health and social needs of migrant construction workers for big sporting events. BMJ. 2021;374:n1591. doi: 10.1136/bmj.n1591 34353809 PMC8340932

[5] Cousins S. Migrant Workers Can’t Afford a Lockdown. Foreign Policy. 2020. https://foreignpolicy.com/2020/04/08/qatar-south-asian-migrant-workers-cant-afford-coronavirus-lockdown-world-cup-2022/

[6] Saric I. Qatar says 400-500 migrant workers died due to World Cup: Axios; 2022. https://www.axios.com/2022/11/29/qatar-migrant-worker-deaths

[7] Pattisson P, McIntyre N, Mukhtar I, Eapen N, Bhuyan MOU, et al. Revealed: 6,500 migrant workers have died in Qatar since World Cup awarded. The Guardian. 2021.

[8] The National Intelligence Council. The future of migration. Washington D.C.; 2021.

[9] Lyu S, Zhu Q, Hu X, Zhu Z, Skitmore M. Migrant workers in the construction industry: A bibliometric and qualitative content analysis. Buildings. 2025;15(5):761. doi: 10.3390/buildings15050761

[10] International Labour Organization. ILO global estimates on international migrant workers – results and methodology. Geneva: International Labour Office; 2018.

[11] OECD. International migration outlook 2020. Paris: OECD; 2020.

[12] Maleyev Y. Construction in the crosshairs: Downside risks via shifts in trade and immigration policy. KPMG Economics; 2025. https://kpmg.com/us/en/articles/2025/april-2025-construction-deep-dive.html

[13] Moyce SC, Schenker M. Migrant workers and their occupational health and safety. Annu Rev Public Health. 2018;39:351–65. doi: 10.1146/annurev-publhealth-040617-013714 29400993

[14] Van der Horst L, Bouaddi O, Williams S, Jago M, Lau K, Furber B, et al. International migrant workers, heat exposure, and climate change: A systematic review of health risks and protective interventions. BMC Glob Public Health. 2025;3(1):107. doi: 10.1186/s44263-025-00224-z 41320785 PMC12667051

[15] Abubakar I, Aldridge RW, Devakumar D, Orcutt M, Burns R, Barreto ML, et al. The UCL-Lancet Commission on migration and health: The health of a world on the move. Lancet. 2018;392(10164):2606–54. doi: 10.1016/S0140-6736(18)32114-7 30528486 PMC7612863

[16] Kennedy S, Kidd MP, McDonald JT, Biddle N. The healthy immigrant effect: Patterns and evidence from four countries. J Int Migr Integr. 2015;16(2):317–32. doi: 10.1007/s12134-014-0340-x

[17] World Health Organization. Global research agenda on health, migration and displacement: strengthening research and translating research priorities into policy and practice. Geneva: World Health Organization; 2023.

[18] International Labour Organization. Forced Labour Convention, 1930 (No. 29). Geneva: International Labour Office; 1930.

[19] Padilla-Frausto DI, Pereira N, Gutiérrez Á, Tan S. Immigrants in California have increased psychological distress and high rates of unmet need for mental health care. Los Angeles, CA: UCLA Center for Health Policy Research; 2023.

[20] Dotsikas K, McGrath M, Osborn DPJ, Walters K, Dykxhoorn J. Exploring the impact of “hostile environment” policies on psychological distress of ethnic groups in the UK: A differences-in-differences analysis. Soc Psychiatry Psychiatr Epidemiol. 2025;60(1):139–48. doi: 10.1007/s00127-024-02705-2 38977506 PMC11790676

[21] Aldridge RW, Nellums LB, Bartlett S, Barr AL, Patel P, Burns R, et al. Global patterns of mortality in international migrants: A systematic review and meta-analysis. Lancet. 2018;392(10164):2553–66. doi: 10.1016/S0140-6736(18)32781-8 30528484 PMC6294735

[22] Hargreaves S, Rustage K, Nellums LB, McAlpine A, Pocock N, Devakumar D, et al. Occupational health outcomes among international migrant workers: A systematic review and meta-analysis. Lancet Glob Health. 2019;7(7):e872–82. doi: 10.1016/S2214-109X(19)30204-9 31122905 PMC6565984

[23] Pega F, Govindaraj S, Tran NT. Health service use and health outcomes among international migrant workers compared with non-migrant workers: A systematic review and meta-analysis. PLoS One. 2021;16(6):e0252651. doi: 10.1371/journal.pone.0252651 34106987 PMC8189512

[24] Lau K, Aldridge R, Norredam M, Mkoma GF, Kugan M, Lin RC-Y, et al. Workplace mortality risk and social determinants among migrant workers: A systematic review and meta-analysis. Lancet Public Health. 2024;9(11):e935–49. doi: 10.1016/S2468-2667(24)00226-3 39486908 PMC11868777

[25] Page MJ, McKenzie JE, Bossuyt PM, Boutron I, Hoffmann TC, Mulrow CD, et al. The PRISMA 2020 statement: An updated guideline for reporting systematic reviews. BMJ. 2021;372:n71. doi: 10.1136/bmj.n71 33782057 PMC8005924

[26] Buller AM, Vaca V, Stoklosa H, Borland R, Zimmerman C. Labour exploitation, trafficking and migrant health: Multi-country findings on the health risks and consequences of migrant and trafficked workers. Geneva: International Organization for Migration and London School of Hygiene and Tropical Medicine; 2015.

[27] Metreau E, Young KE, Eapen SG. World Bank country classifications by income level for 2024-2025. Washington DC: World Bank; 2024. https://blogs.worldbank.org/en/opendata/world-bank-country-classifications-by-income-level-for-2024-2025

[28] Cha S, Cho Y. Fatal and non-fatal occupational injuries and diseases among migrant and native workers in South Korea. Am J Ind Med. 2014;57(9):1043–52. doi: 10.1002/ajim.22349 24953056

[29] Wu TN, Liou SH, Hsu CC, Chao SL, Liou SF, Ko KN, et al. Epidemiologic study of occupational injuries among foreign and native workers in Taiwan. Am J Ind Med. 1997;31(5):623–30. doi: 10.1002/(SICI)1097-0274(199705)31:5<623::AID-AJIM18>3.0.CO;2-0 9099366

[30] Dong XS, Choi SD, Borchardt JG, Wang X, Largay JA. Fatal falls from roofs among U.S. construction workers. J Safety Res. 2013;44:17–24. doi: 10.1016/j.jsr.2012.08.024 23398700

[31] Dong XS, Fujimoto A, Ringen K, Men Y. Fatal falls among Hispanic construction workers. Accid Anal Prev. 2009;41(5):1047–52. doi: 10.1016/j.aap.2009.06.012 19664444

[32] Dong XS, Wang X, Largay JA, Sokas R. Long-term health outcomes of work-related injuries among construction workers--findings from the National Longitudinal Survey of Youth. Am J Ind Med. 2015;58(3):308–18. doi: 10.1002/ajim.22415 25678458

[33] Dong XS, West GH, Holloway-Beth A, Wang X, Sokas RK. Heat-related deaths among construction workers in the United States. Am J Ind Med. 2019;62(12):1047–57. doi: 10.1002/ajim.23024 31328819

[34] Corvalan CF, Driscoll TR, Harrison JE. Role of migrant factors in work-related fatalities in Australia. Scand J Work Environ Health. 1994;20(5):364–70. doi: 10.5271/sjweh.1385 7863300

[35] Salvatore MA, Baglio G, Cacciani L, Spagnolo A, Rosano A. Work-related injuries among immigrant workers in Italy. J Immigr Minor Health. 2013;15(1):182–7. doi: 10.1007/s10903-012-9673-8 22752688

[36] Nuwayhid I, Fayad R, Tamim H, Kassak K, Khogali M. Work-related injuries in Lebanon: Does nationality make a difference? Am J Ind Med. 2003;44(2):172–81. doi: 10.1002/ajim.10246 12874850

[37] Zhang X, Yu S, Wheeler K, Kelleher K, Stallones L, Xiang H. Work-related non-fatal injuries among foreign-born and US-born workers: Findings from the U.S. National Health Interview Survey, 1997-2005. Am J Ind Med. 2009;52(1):25–36. doi: 10.1002/ajim.20642 18942664

[38] Byler CG. Hispanic/Latino fatal occupational injury rates. Mon Labor Rev. 2013; 136(2):14-23.

[39] Byler CG, Robinson WC. Differences in patterns of mortality between foreign-born and native-born workers due to fatal occupational injury in the USA from 2003 to 2010. J Immigr Minor Health. 2018;20(1):26–32. doi: 10.1007/s10903-016-0503-2 27704387

[40] Cruz Y, Bunn TL, Hanner N, Slavova S. Characterization of foreign-born vs. native-born worker fatalities in Kentucky, 2001-2014. J Immigr Minor Health. 2018;20(2):448–55. doi: 10.1007/s10903-017-0550-3 28197863

[41] Giraudo M, Bena A, Costa G. Migrant workers in Italy: An analysis of injury risk taking into account occupational characteristics and job tenure. BMC Public Health. 2017;17(1):351. doi: 10.1186/s12889-017-4240-9 28431498 PMC5401389

[42] Giraudo M, Bena A, Mosca M, Farina E, Leombruni R, Costa G. Differences in work injury risk between immigrants and natives: Changes since the economic recession in Italy. BMC Public Health. 2019;19(1):836. doi: 10.1186/s12889-019-7178-2 31248410 PMC6598376

[43] Jeong BY. Prevalence of occupational accidents and factors associated with deaths and disabilities in the shipbuilding industry: Comparisons of novice and skilled workers. Work. 2021;69(3):997–1005. doi: 10.3233/WOR-213530 34219693

[44] Konda S, Tiesman HM, Reichard AA. Fatal traumatic brain injuries in the construction industry, 2003-2010. Am J Ind Med. 2016;59(3):212–20. doi: 10.1002/ajim.22557 26765167

[45] Scrimshaw S, Baker R, Platner J, Shaw-Salazar L. Safety is seguridad: a workshop summary. Washington DC: The National Academies Press; 2003. doi: 10.17226/10641

[46] Steege AL, Baron SL, Marsh SM, Menéndez CC, Myers JR. Examining occupational health and safety disparities using national data: A cause for continuing concern. Am J Ind Med. 2014;57(5):527–38. doi: 10.1002/ajim.22297 24436156 PMC4556419

[47] Jung S, Kim T-K, Kim Y-J, Kim Y-K, Kang D, Kim S-Y. Epidemiology of occupational injuries in construction workers between 2009 and 2018 in South Korea. Am J Ind Med. 2023;66(2):155–66. doi: 10.1002/ajim.23448 36433706 PMC10100143

[48] King TL, Alfonzo LF, LaMontagne AD, Maheen H. Variations in suicide rates among Australian male construction workers by country of birth. Lancet Reg Health West Pac. 2024;49:101155. doi: 10.1016/j.lanwpc.2024.101155 39381020 PMC11459016

[49] Frickmann F, Wurm B, Jeger V, Lehmann B, Zimmermann H, Exadaktylos AK. 782 consecutive construction work accidents: who is at risk? A 10-year analysis from a Swiss university hospital trauma unit. Swiss Med Wkly. 2012;142:w13674. doi: 10.4414/smw.2012.13674 22956176

[50] Connell PP, Saddak T, Harrison I, Kelly S, Bobart A, McGettrick P, et al. Construction-related eye injuries in Irish nationals and non-nationals: Attitudes and strategies for prevention. Ir J Med Sci. 2007;176(1):11–4. doi: 10.1007/s11845-007-0002-5 17849517

[51] Tuma MA, Acerra JR, El-Menyar A, Al-Thani H, Al-Hassani A, Recicar JF, et al. Epidemiology of workplace-related fall from height and cost of trauma care in Qatar. Int J Crit Illn Inj Sci. 2013;3(1):3–7. doi: 10.4103/2229-5151.109408 23724377 PMC3665115

[52] Elders LAM, Burdorf A, Ory FG. Ethnic differences in disability risk between Dutch and Turkish scaffolders. J Occup Health. 2004;46(5):391–7. doi: 10.1539/joh.46.391 15492456

[53] Dong XS, Wang X, Largay JA, Platner JW, Stafford E, Cain CT, et al. Fatal falls in the U.S. residential construction industry. Am J Ind Med. 2014;57(9):992–1000. doi: 10.1002/ajim.22341 24890625

[54] Yanai O, Goldin L, Kugel C, Hiss J. Occupational fatalities in Israel. J Clin Forensic Med. 1999;6(3):129–32. doi: 10.1016/s1353-1131(99)90058-5 15335480

[55] Davidson CC, Orr DJ. Occupational injuries in foreign-national workers presenting to St James’s Hospital Plastic Surgery service. Ir Med J. 2009;102(4):108–10. 19552289

[56] Forst L, Avila S, Anozie S, Rubin R. Traumatic occupational injuries in Hispanic and foreign born workers. Am J Ind Med. 2010;53(4):344–51. doi: 10.1002/ajim.20748 19753594

[57] Arndt V, Rothenbacher D, Zschenderlein B, Schuberth S, Brenner H. Body mass index and premature mortality in physically heavily working men--a ten-year follow-up of 20,000 construction workers. J Occup Environ Med. 2007;49(8):913–21. doi: 10.1097/JOM.0b013e31814544fe 17693790

[58] Capasso R, Zurlo MC, Smith AP. Ethnicity, work-related stress and subjective reports of health by migrant workers: A multi-dimensional model. Ethn Health. 2018;23(2):174–93. doi: 10.1080/13557858.2016.1258041 27846733

[59] Chung JW-Y, Wong BY-M, Yan VC-M, Chung LM-Y, So HC-F, Chan A. Cardiovascular health of construction workers in Hong Kong: A cross-sectional study. Int J Environ Res Public Health. 2018;15(6):1251. doi: 10.3390/ijerph15061251 29895813 PMC6025116

[60] Korkmaz S, Park DJ. Comparison of safety perception between foreign and local workers in the construction industry in Republic of Korea. Saf Health Work. 2018;9(1):53–8. doi: 10.1016/j.shaw.2017.07.002 30363074 PMC6111130

[61] Pawthaisong K, Jongprasithporn M, Vongpisal C, Yodpijit N. Risk assessment among Thai and foreign workers in construction companies. In: 2018 IEEE International Conference on Industrial Engineering and Engineering Management (IEEM); 2018 Dec 16–19; Bangkok, Thailand. IEEE; 2018. p. 103–7. 10.1109/IEEM.2018.8607328.

[62] Arndt V, Rothenbacher D, Daniel U, Zschenderlein B, Schuberth S, Brenner H. All-cause and cause specific mortality in a cohort of 20 000 construction workers; results from a 10 year follow up. Occup Environ Med. 2004;61(5):419–25. doi: 10.1136/oem.2003.008680 15090662 PMC1740777

[63] Arndt V, Rothenbacher D, Daniel U, Zschenderlein B, Schuberth S, Brenner H. Construction work and risk of occupational disability: a ten year follow up of 14,474 male workers. Occup Environ Med. 2005;62(8):559–66. doi: 10.1136/oem.2004.018135 16046609 PMC1741071

[64] Ricci F, Bravo G, Modenese A, De Pasquale F, Ferrari D, Bello M, et al. Risk perception and ethnic background in construction workers: Results of a cross-sectional study in a group of trainees of a vocational school in Italy. Eur J Investig Health Psychol Educ. 2021;11(1):96–109. doi: 10.3390/ejihpe11010008 34542452 PMC8314337

[65] Salehi SH, Azad YS, Bagheri T, Ghadimi T, Rahbar A, Ehyaei P, et al. Epidemiology of occupational electrical injuries. J Burn Care Res. 2022;43(2):399–402. doi: 10.1093/jbcr/irab171 34562012

[66] Ahonen EQ, Benavides FG, Benach J. Immigrant populations, work and health--a systematic literature review. Scand J Work Environ Health. 2007;33(2):96–104. doi: 10.5271/sjweh.1112 17460797

[67] De Jesus-Rivas M, Conlon HA, Burns C. The impact of language and culture diversity in occupational safety. Workplace Health Saf. 2016;64(1):24–7. doi: 10.1177/2165079915607872 26800895

[68] Lall R, Ravindranath D. The case of female workers in India’s construction sector. Barcelona: United Cities and Local Governments; 2022.

[69] Manjunath S, Shashi Kumar M, Minu A. Literacy and livelihood of women migrant construction workers: An analytical study. J Int Women’s Stud. 2021;22(6): 76–83.

[70] International Labour Organization. ILO global estimates on international migrant workers: international migrants in the labour force. Geneva: International Labour Organization; 2024.

[71] Valles SA. The challenges of choosing and explaining a phenomenon in epidemiological research on the “Hispanic Paradox”. Theor Med Bioeth. 2016;37(2):129–48. doi: 10.1007/s11017-015-9349-1 26754488

[72] Zimmerman C, Kiss L. Human trafficking and exploitation: A global health concern. PLoS Med. 2017;14(11):e1002437. doi: 10.1371/journal.pmed.1002437 29166396 PMC5699819

[73] United Nations. Transforming our world: The 2030 agenda for sustainable development. New York: United Nations; 2015.

[74] World Bank. World development report 2023: Migrants, refugees, and societies. Washington DC, USA: World Bank; 2023.

[75] de Haas H. Migration and development: A theoretical perspective. Int Migr Rev. 2010;44(1):227–64. 10.1111/j.1747-7379.2009.00804.x PMC4744987 26900199

[76] United Nations General Assembly. Global compact for safe, orderly and regular migration. New York: United Nations; 2018.

[77] Grittner AM, Johnson MS. When labor enforcement and immigration enforcement collide: Deterring worker complaints worsens workplace safety. Kalamazoo, Michigan: W.E. Upjohn Institute for Employment Research; 2021. Upjohn Institute Working Paper 21-353.

[78] Reyes EA, Plevin R. Immigration crackdown could deter workers from reporting hazards, advocates warn. Los Angeles Times. 2025.

[79] Dong XS, Wang X, Daw C. Fatal and nonfatal injuries among Hispanic construction workers, 1992-2008. CPWR- The Center for Construction Research and Training; 2010.

[80] Vargas ED, Sanchez GR, Juarez M. Fear by association: Perceptions of anti-immigrant policy and health outcomes. J Health Polit Policy Law. 2017;42(3):459–83. 10.1215/03616878-3802940 PMC6624117 28213396

[81] Hiam L, Steele S, McKee M. Creating a “hostile environment for migrants”: the British government’s use of health service data to restrict immigration is a very bad idea. Health Econ Policy Law. 2018;13(2):107–17. doi: 10.1017/S1744133117000251 29306349

[82] Berlin A, Koski-Karell V, Page KR, Polk S. The right and left hands of the state - two patients at risk of deportation. N Engl J Med. 2019;381(3):197–201. doi: 10.1056/NEJMp1811607 31314963

[83] Pattisson P, Acharya P. Revealed: migrant workers in Qatar forced to pay billions in recruitment fees. The Guardian. 2022.

[84] Robinson K. What is the kafala system? New York: Council on Foreign Relations; 2022. https://www.cfr.org/backgrounder/what-kafala-system

[85] Lyu S, Hon CKH, Chan APC, Gao R, Skitmore M, Hu X. Comparing the perceptions of ethnic minority construction workers and management staff on the factors affecting safety communications. J Safety Res. 2025;92:511–21. doi: 10.1016/j.jsr.2024.11.029 39986870

[86] Mehany MSHM, Fisher GG, Thiese M, Kumar S. Assessment of construction workers’ mental health to improve wellbeing. Silver Spring, MD: CPWR — The Center for Construction Research and Training; 2024.

[87] Amoadu M, Ansah EW, Sarfo JO, Hormenu T. Impact of climate change and heat stress on workers’ health and productivity: A scoping review. J Clim Change Health. 2023;12:100249. doi: 10.1016/j.joclim.2023.100249

[88] Amnesty International. In the prime of their lives: Qatar’s failure to investigate, remedy and prevent migrant workers’ deaths. London: Amnesty International; 2021.

[89] Pradhan B, Kjellstrom T, Atar D, Sharma P, Kayastha B, Bhandari G, et al. Heat stress impacts on cardiac mortality in Nepali migrant workers in Qatar. Cardiology. 2019;143(1—2):37–48. doi: 10.1159/000500853 31302648

[90] World Health Organization, World Meteorological Organization. Climate change and workplace heat stress: technical report and guidance. Geneva: World Health Organization; 2025. 10.1177/10482911251390889 PMC12804399 41442273

