# Supplementary material for: Occupational health outcomes among migrant and non-migrant construction workers: A systematic review and narrative synthesis
Source: PLOS Glob Public Health. 2026 Nov 13;6(11):e0007624. doi: 10.1371/journal.pgph.0007624 (PMC13766298; doi:10.1371/journal.pgph.0007624)
Supplement: Supplementary file 2 [file pgph.0007624.s003.docx]

# S1 Text. Search Terms

*The following search strategies were applied across 14 electronic databases and grey literature sources. All searches were restricted to studies published between January 1,1990 and May 15, 2025. Full adapted search strings for all included databases are available upon request from the corresponding author.*

**Database 1 Example: Web of Science Search Strategy**

**#1** ((TS=(labo*rer*)) OR TS=(worker*)) NOT (SILOID==("PPRN"))

**#2** (((((((((((((((((((((((((((((((((((( TS=("transients and migrants")) OR TS=(refugee*)) OR TS=(migrant*)) OR TS=(migrat*)) OR TS=(immigrant*)) OR TS=(immigrat*)) OR TS=(asylum NEAR seeker*)) OR TS=(transient*)) OR TS=(ethnic*)) OR TS=(displaced NEAR person*)) OR TS=(ethnic NEAR minorit*)) OR TS=(illegal NEAR migrant*)) OR TS=(illegal NEAR migrat*)) OR TS=(migrant NEAR worker*)) OR TS=(nomad*)) OR TS=(minority NEAR group*)) OR TS=(nationalit*)) OR TS=(labo*r NEAR migrant*)) OR TS=(labo*r NEAR migrat*)) OR TS=(foreign NEAR worker*)) OR TS=(minorit*)) OR TS=(labo*r NEAR trafficking)) OR TS=(forced NEAR labo*r)) OR TS=("human trafficking")) OR TS=("people trafficking")) OR TS=("child trafficking")) OR TS=("trafficking in people")) OR TS=("victim of trafficking")) OR TS=("survivor of trafficking")) OR TS=("trafficking for" NEAR labo*r NEAR exploitation)) OR TS=("trafficked people")) OR TS=("trafficked women")) OR TS=("trafficked men")) OR TS=("trafficked children")) OR TS=(trafficked NEAR person*))) NOT (SILOID==("PPRN"))

**#3**	((((((((((((((((((((((((((((((((((((((((((( TS=("construction industry")) OR TS=(construction*)) OR TS=("building industry")) OR TS=(building*)) OR TS=(builder*)) OR TS=(labo*rer*)) OR TS=(worker*)) OR TS=(contractor*)) OR TS=(supervisor*)) OR TS=(machine NEAR driver*)) OR TS=(machine NEAR operator*)) OR TS=(brickmason*)) OR TS=("pile driver"*)) OR TS=(concrete NEAR worker*)) OR TS=(metal NEAR worker*)) OR TS=(road NEAR builder*)) OR TS=(pipe NEAR driver*)) OR TS=(tower NEAR crane*)) OR TS=(fitter*)) OR TS=(carpenter*)) OR TS=(rammer*)) OR TS=(scaffolder*)) OR TS=(bricklayer*)) OR TS=(pointer*)) OR TS=(plasterer*)) OR TS=(plasterpainter*)) OR TS=(roofer*)) OR TS=(plumber*)) OR TS=(glazier*)) OR TS=(glasier*)) OR TS=(screeder*)) OR TS=(electrician*)) OR TS=(tiler*)) OR TS=(painter*)) OR TS=(paviour*)) OR TS=(pavier*)) OR TS=(ironwork*)) OR TS=(metalwork*)) OR TS=(asphalt*)) OR TS=(roofing*)) OR TS=("construction materials")) OR TS=("facility design and construction")) OR TS=(painting)) OR TS=(construction NEAR material*)

**#4**	(((((((((((((((((TS=("enviornmental exposure")) OR TS=("hazardous substances")) OR TS=(infection)) OR TS=("communicable diseases")) OR TS=("disease outbreaks")) OR TS=(occupation*)) OR TS=(work NEAR related*)) OR TS=(work*)) OR TS=(employee*)) OR TS=(labo*r*)) OR TS=(job*)) OR TS=(employment*)) OR TS=("occupational medicine")) OR TS=("occupational exposure")) OR TS=("environmental exposure")) OR TS=("accidents, occupational")) OR TS=(occupational NEAR disease*)) AND TI=(influenza OR hiv OR aids OR physical therapy OR biomechanic* OR tobacco OR smoking OR diet* OR emergency)

**#5**	(((((((((((((((((((((((((((((((((((((((((TS=(*health)) OR TS=(*health NEAR status)) OR TS=(*health NEAR "status indicators")) OR TS=(*physical NEAR fitness)) OR TS=(*mens NEAR health)) OR TS=(*womens NEAR health)) OR TS=(*attitude NEAR "to health")) OR TS=(*disease)) OR TS=(*chronic NEAR disease)) OR TS=(*sleep NEAR disorders)) OR TS=(aging)) OR TS=(*mental NEAR health)) OR TS=(*mental NEAR disorders)) OR TS=(*adaptation NEAR psychological)) OR TS=(*self NEAR concept)) OR TS=(*personal NEAR satisfaction)) OR TS=(*stress NEAR psychological)) OR TS=(*stress NEAR physiological)) OR TS=(*depression)) OR TS=(*depressive NEAR disorder)) OR TS=(*anxiety)) OR TS=(*anxiety NEAR disorders)) OR TS=("employee health")) OR TS=("industrial hygiene")) OR TS=("occupational health")) OR TS=("occupational safety")) OR TS=("worker health")) OR TS=("occupational injuries")) OR TS=("occupational injury")) OR TS=("occupational disease")) OR TS=(work NEAR injur*)) OR TS=(disability)) OR TS=("return to work")) OR TS=("workers compensation")) OR TS=("prediction of work disability")) OR TS=("prediction of return to work")) OR TS=(death*)) OR TS=(fatal)) OR TS=(fatalit*)) OR TS=(mortality)) OR TS=(morbidity)) AND TI=((health OR "ill health" OR "well NEAR being" OR disease* OR disorders OR complaints OR psychosocial OR psychological OR sleep OR mental OR depression OR anxiety OR stress OR distress OR ageing OR aging) NOT "oxidative stress")

**#6**	#1 AND #2 AND #4 AND #5

**#7**	#2 AND #3 AND #4 AND #5

**#8**	#6 OR #7

**Database 2 Example: APA Psych**

(((('construction industry'/exp OR 'construction industry') OR construction* OR 'building industry' OR building* OR builder* OR labo*rer* OR contractor* OR supervisor* OR 'machine driver*' OR 'machine operator*' OR brickmason* OR 'pile driver*' OR 'concrete worker*' OR 'metal worker*' OR 'road builder*' OR 'pipe driver*' OR 'tower crane*' OR fitter* OR carpenter* OR rammer* OR scaffolder* OR bricklayer* OR pointer* OR plasterer* OR plasterpainter* OR roofer* OR plumber* OR glazier* OR glasier* OR screeder* OR electrician* OR tiler* OR painter* OR paviour* OR pavier* OR ironwork* OR metalwork* OR asphalt* OR roofing OR 'construction materials'/exp OR 'facility design and construction' OR painting OR 'construction material*') AND ('transients and migrants'/exp OR refugee* OR migrant* OR migrat* OR immigrant* OR immigrat* OR transient* OR ethnic* OR 'displaced person*' OR 'ethnic minorit*' OR 'illegal migrant*' OR 'illegal migrat*' OR 'migrant worker*' OR nomad* OR 'minority group*' OR minorit* OR nationalit* OR 'labo*r migrant*' OR 'labo*r migrat*' OR 'foreign worker*' OR 'labo*r trafficking' OR 'forced labo*r' OR 'human trafficking' OR 'people trafficking' OR 'child trafficking' OR 'trafficking in people' OR 'victim of trafficking' OR 'survivor of trafficking' OR 'trafficking for labo*r exploitation' OR 'trafficked people' OR 'trafficked women' OR 'trafficked men' OR 'trafficked children' OR 'trafficked person') AND (('environmental exposure' OR 'hazardous substances' OR 'infection' OR 'communicable diseases' OR 'disease outbreaks' OR influenza:ti OR hiv:ti OR aids:ti OR 'physical therapy':ti OR biomechanic*:ti OR tobacco:ti OR smoking:ti OR diet*:ti OR emergency:ti) OR occupations OR occupation* OR 'work related*' OR work* OR employee* OR work OR labo*r* OR job* OR employment OR employment* OR 'occupational medicine' OR 'occupational exposure' OR 'environmental exposure' OR 'accidents, occupational'/exp OR 'occupational medicine'/exp OR 'occupational disease*') AND (((health:ti OR 'ill health':ti OR 'well being':ti OR disease*:ti OR disorders:ti OR complaints:ti OR psychosocial:ti OR psychological:ti OR sleep:ti OR mental:ti OR depression:ti OR anxiety:ti OR stress:ti OR distress:ti OR ageing:ti OR aging:ti) NOT 'oxidative stress':ti) OR ('health' OR 'health status' OR 'health status indicators' OR 'physical fitness' OR 'mens health' OR 'womens health' OR 'attitude to health' OR 'disease' OR 'chronic disease' OR 'sleep disorders' OR aging) OR ('mental health' OR 'mental disorders' OR 'adaptation, psychological' OR 'self concept' OR 'personal satisfaction' OR 'stress, psychological'/exp OR 'stress, physiological' OR depression OR 'depressive disorder' OR anxiety OR 'anxiety disorders') OR 'employee health' OR 'industrial hygiene' OR 'occupational health'/exp OR 'occupational health' OR 'occupational safety' OR 'worker health' OR 'occupational injuries'/exp OR 'occupational injury' OR 'occupational disease' OR 'work injur*' OR disability OR 'return to work' OR 'workers compensation' OR 'prediction of work disability' OR 'prediction of return to work' OR death* OR fatal OR fatalit* OR mortality OR morbidity)) OR (('transients and migrants'/exp OR refugee* OR migrant* OR migrat* OR immigrant* OR immigrat* OR transient* OR ethnic* OR 'displaced person*' OR 'ethnic minorit*' OR 'illegal migrant*' OR 'illegal migrat*' OR 'migrant worker*' OR nomad* OR 'minority group*' OR minorit* OR nationalit* OR 'labo*r migrant*' OR 'labo*r migrat*' OR 'foreign worker*' OR 'labo*r trafficking' OR 'forced labo*r' OR 'human trafficking' OR 'people trafficking' OR 'child trafficking' OR 'trafficking in people' OR 'victim of trafficking' OR 'survivor of trafficking' OR 'trafficking for labo*r exploitation' OR 'trafficked people' OR 'trafficked women' OR 'trafficked men' OR 'trafficked children' OR 'trafficked person') AND (('environmental exposure' OR 'hazardous substances' OR 'infection' OR 'communicable diseases' OR 'disease outbreaks' OR influenza:ti OR hiv:ti OR aids:ti OR 'physical therapy':ti OR biomechanic*:ti OR tobacco:ti OR smoking:ti OR diet*:ti OR emergency:ti) OR occupations OR occupation* OR 'work related*' OR work* OR employee* OR work OR labo*r* OR job* OR employment OR employment* OR 'occupational medicine' OR 'occupational exposure' OR 'environmental exposure' OR 'accidents, occupational'/exp OR 'occupational medicine'/exp OR 'occupational disease*') AND (((health:ti OR 'ill health':ti OR 'well being':ti OR disease*:ti OR disorders:ti OR complaints:ti OR psychosocial:ti OR psychological:ti OR sleep:ti OR mental:ti OR depression:ti OR anxiety:ti OR stress:ti OR distress:ti OR ageing:ti OR aging:ti) NOT 'oxidative stress':ti) OR ('health' OR 'health status' OR 'health status indicators' OR 'physical fitness' OR 'mens health' OR 'womens health' OR 'attitude to health' OR 'disease' OR 'chronic disease' OR 'sleep disorders' OR aging) OR ('mental health' OR 'mental disorders' OR 'adaptation, psychological' OR 'self concept' OR 'personal satisfaction' OR 'stress, psychological'/exp OR 'stress, physiological' OR depression OR 'depressive disorder' OR anxiety OR 'anxiety disorders') OR 'employee health' OR 'industrial hygiene' OR 'occupational health'/exp OR 'occupational health' OR 'occupational safety' OR 'worker health' OR 'occupational injuries'/exp OR 'occupational injury' OR 'occupational disease' OR 'work injur*' OR disability OR 'return to work' OR 'workers compensation' OR 'prediction of work disability' OR 'prediction of return to work' OR death* OR fatal OR fatalit* OR mortality OR morbidity) AND (labo*rer* OR worker*)))

AND restricted to xx/xx/xxx [DATE] to xx/xx/xxx [DATE]
