# Supplementary material for: Occupational health outcomes among migrant and non-migrant construction workers: A systematic review and narrative synthesis
Source: PLOS Glob Public Health. 2026 Nov 13;6(11):e0007624. doi: 10.1371/journal.pgph.0007624 (PMC13766298; doi:10.1371/journal.pgph.0007624)
Supplement: Supplementary file 3 [file pgph.0007624.s004.docx]

# S2 Text. Definitions of Key Search Terms in Inclusion Criteria.

| Term | Definition |
| --- | --- |
| Construction Worker | A construction worker is deﬁned as follows: a person “working at a construction site for building/housing/residential or road/highway/civil engineering or offices/commercial or industrial installation (for example, ventilation, pipelines and siding) work” (1). Construction work undertaken by a worker involves the creation, addition, or modification of structures, as well as continuous maintenance and repairs. Other areas of construction are “refurbishment and demolition of buildings and engineering projects as well as plumbing, heating, ventilation and air conditioning work” (2). The deﬁnition of construction worker is based on the North American Industry Classiﬁcation System and a 2013 Cochrane review on construction work (1, 2). |
| Migrant Worker | A migrant worker is defined as someone who has moved “away from their usual place of residence to a new place of residence, either across an international border or within a State” as defined by the International Organization for Migration (3).  In this category, we have included workers that have been subject to labor trafficking, as defined below. Although some organizations define migrant status based on their country of origin, this review included all non-nationals, foreign-born, and undocumented workers, as well as internal or domestic migrants. |
| Labor Trafficking | Labor trafficking is defined as the recruitment and movement of an individual, by a variety of means, for the purpose of labor exploitation. This definition is an adaptation of the United Nations Optional Protocol on the Prevention, Punishment and Suppression of Trafficking in Persons definition for human trafficking (4). |
| Occupational Exposures | An occupational exposure is defined as an exposure to a chemical, physical, biological, or social agent that occurs as a result of occupational factors (5). The definition is derived from the International Labour Organization’s Encyclopaedia of Occupational Safety & Health (6). |
| Negative Health Outcome | Negative health outcomes are categorized according to our conceptual model to include occupational and cause-specific injuries, morbidity, mortality; all-cause mortality; and occupational diseases involving multiple organ systems, such as infectious disease, oncologic, and mental health (7, 8, and 9). |

References

1.	U.S. Census Bureau. North American Industry Classification System- Sector 23: Construction 2022 [Available from: <http://www.census.gov/eos/www/naics/>.

2.	van der Molen HF, Basnet P, Hoonakker PL, Lehtola MM, Lappalainen J, Frings-Dresen MH, et al. Interventions to prevent injuries in construction workers. Cochrane Database Syst Rev. 2018;2(2):CD006251.

3.	International Organization for Migration. World Migration Report 2020. Geneva: International Organization for Migration; 2020.

4.	United Nations. United Nations Convention against Transnational Organized Crime and the Protocols. New York: United Nations; 2000.

5.	Haagsma JA, Tariq L, Heederik DJ, Havelaar AH. Infectious disease risks associated with occupational exposure: a systematic review of the literature. Occup Environ Med. 2012;69(2):140–6.

6.	International Labour Organization. Encyclopaedia of Occupational Health and Safety. Geneva: International Labour Organization; 2012.

7.	OSHA Health Hazards in Construction Workbook: Construction Safety Council; 2012. Available from: [https://www.osha.gov/dte/grant_materials/fy09/sh-19495-09/health_hazards_workbook.pdf](https://www.google.com/url?q=https://www.osha.gov/dte/grant_materials/fy09/sh-19495-09/health_hazards_workbook.pdf&sa=D&source=docs&ust=1779330415897446&usg=AOvVaw2anqtDLXqQXtP9XoykOFfD).

8.	Construction. Encyclopedia of Occupational Safety & Health1998.

9. Lim SS, Vos T, Flaxman AD, Danaei G, Shibuya K, Adair-Rohani H, et al. A comparative risk assessment of burden of disease and injury attributable to 67 risk factors and risk factor clusters in 21 regions, 1990-2010: a systematic analysis for the Global Burden of Disease Study 2010. Lancet. 2012;380(9859):2224-60
