# Supplementary material for: Occupational health outcomes among migrant and non-migrant construction workers: A systematic review and narrative synthesis
Source: PLOS Glob Public Health. 2026 Nov 13;6(11):e0007624. doi: 10.1371/journal.pgph.0007624 (PMC13766298; doi:10.1371/journal.pgph.0007624)
Supplement: Supplementary file 4 [file pgph.0007624.s005.docx]

# S3 Text. Study Selection

*Inclusion Criteria*

**Population:**  The review included studies that address males or females (adults or children) who are migrant and non-migrant construction workers.  No lower or upper age limits were used.  Children are defined as individuals aged under 18 years of age.  Ethnic groups (e.g. Hispanics/Latinos in the United States) were included only when they were explicitly identified as foreign-born migrants. Studies of day-laborers or low-skilled, unskilled, blue collar, manual laborers were not considered specific enough and were excluded. Studies with no data regarding non-migrant construction workers were also excluded.

**Outcomes:** The review included studies that reported occupational health outcomes related to construction work for both migrant and non-migrant workers.

**Study designs:** The review included papers using a range of study designs, including peer-reviewed randomised controlled trials (RCTs) or other experimental studies; cohort studies; case control studies; cross-sectional surveys; secondary analyses of organisational records; case series analyses; case studies; qualitative studies; review articles; or grey literature.

*Restrictions:*

**Date of publication:** Because construction technologies and equipment have changed significantly in recent history, the review searched multiple electronic databases from January 1^st^, 1990 up to May 15^th^, 2025.

**Language:** No language restrictions were used.

**Setting:** Country setting was not be limited

*Bias:*

**Publication, location and language bias:** We acknowledge that studies that report statistically significant results are more likely to be published than those reporting no significance. Furthermore, these studies are more likely to be published in a journal that is indexed in an electronic database, to be published in English, and to be cited more often. In addition, research reported in a language other than English is less likely to be published in an indexed journal.  We have included studies written in languages other than English to reduce this particular bias. Also, by searching a breadth of electronic databases and calling upon expert recommendations to identify in-press or un-indexed articles, we may further minimize publication and location bias.

**Duplication bias:** In cases where our search yielded multiple eligible papers from the same study, the paper with the largest sample was included.
