# Supplementary material for: Occupational health outcomes among migrant and non-migrant construction workers: A systematic review and narrative synthesis
Source: PLOS Glob Public Health. 2026 Nov 13;6(11):e0007624. doi: 10.1371/journal.pgph.0007624 (PMC13766298; doi:10.1371/journal.pgph.0007624)
Supplement: Supplementary file 5 [file pgph.0007624.s007.docx]

**Response to Reviewers**

**Journal Requirements:**

1. Please amend your detailed Financial Disclosure statement. This is published with the article. It must therefore be completed in full sentences and contain the exact wording you wish to be published.

Please clarify all sources of financial support for your study. List the grants, grant numbers, and organizations that funded your study, including funding received from your institution. Please note that suppliers of material support, including research materials, should be recognized in the Acknowledgements section rather than in the Financial Disclosure.

State the initials, alongside each funding source, of each author to receive each grant. For example: "This work was supported by the National Institutes of Health (####### to AM; ###### to CJ) and the National Science Foundation (###### to AM)."

State what role the funders took in the study. If the funders had no role in your study, please state: “The funders had no role in study design, data collection and analysis, decision to publish, or preparation of the manuscript.”

If any authors received a salary from any of your funders, please state which authors and which funders.

*Thank you for your feedback. We have revised the language to properly capture the financial support received for this study. The funding statement now reads (see lines 61-63):*

*This work was supported by the United States Department of State (Award No. S-SGTIP-11-GR-0036 to HS). The funders had no role in study design, data collection and analysis, decision to publish, or preparation of manuscript.*

2. We note that your Data Availability Statement is currently as follows: All relevant data regarding the systematic review is including within the paper and Supporting Information files.

Please confirm at this time whether or not your submission contains all raw data required to replicate the results of your study. Authors must share the “minimal data set” for their submission. PLOS defines the minimal data set to consist of the data required to replicate all study findings reported in the article, as well as related metadata and methods (<https://journals.plos.org/plosone/s/data-availability#loc-minimal-data-set-definition>).

For example, authors should submit the following data:

- The values behind the means, standard deviations and other measures reported;
- The values used to build graphs;
- The points extracted from images for analysis.

Authors do not need to submit their entire data set if only a portion of the data was used in the reported study.

If your submission does not contain these data, please either upload them as Supporting Information files or deposit them to a stable, public repository and provide us with the relevant URLs, DOIs, or accession numbers. For a list of recommended repositories, please see <https://journals.plos.org/plosone/s/recommended-repositories>.

 If there are ethical or legal restrictions on sharing a de-identified data set, please explain them in detail (e.g., data contain potentially sensitive information, data are owned by a third-party organization, etc.) and who has imposed them (e.g., an ethics committee). Please also provide contact information for a data access committee, ethics committee, or other institutional body to which data requests may be sent. If data are owned by a third party, please indicate how others may request data access.

*We thank the editor for raising this important point. We confirm that our submission contains the minimum dataset required to replicate the findings of this systematic review. The search strategy, eligibility criteria, selection process, and extracted data are included in the manuscript and supporting files.*

*Tables 2-5 present the extracted data and measures of association used in the synthesis. As this is systematic review with narrative synthesis, no primary data collection was undertaken. We have revised the Data Availability statement accordingly.*

*Our revised data availability statement is as follows:*

*This submission contains all the data required to replicate the findings of the review. The minimal dataset consists of the search strategy, eligibility criteria and extracted data from included studies, all of which are provided in the manuscript and supporting files. Extracted data are presented in Tables 2-5. As this study is a systematic review with narrative synthesis of previously published studies, no new primary data were collected. Full raw data sets of the original studies are not available to us, as they remain the property of the original research teams.*

3. Please provide separate figure files in .tif or .eps format.

For more information about figure files please see our guidelines:

<https://journals.plos.org/globalpublichealth/s/figures>
<https://journals.plos.org/globalpublichealth/s/figures#loc-file-requirements>

*Thank you. We have converted the format of our figures and attached them in the .tif format.*

4. We have noticed that you have uploaded Supporting Information files, but you have not included a list of legends. Please add a full list of legends for your Supporting Information files after the references list.

*Thank you for this feedback. We have now included legends for our Supporting Information files after the reference list. We have added additional files to our Supporting Information.*

5. As required by our policy on Data Availability, please ensure your manuscript or supplementary information includes the following:

*Thank you for this guidance. We have carefully reviewed each element of this requirement. We have included responses to each point below in green:*

5.1. A numbered table of all studies identified in the literature search, including those that were excluded from the analyses.
5.2. For every excluded study, the table should list the reason(s) for exclusion.

*Points 5.1-5.2 (numbered table of all studies with exclusion reasons): We respectfully note that including a full list of (in this case 1,972) full-texts assessed for eligibility with individual exclusion reasons is not always standard practice in all systematic reviews published in PLOS Global Public Health. We reviewed recently published systematic reviews in this journal and found many that did not include study-by-study exclusion table for full-texts [for example:* [*https://doi.org/10.1371/journal.pgph.0005196*](https://doi.org/10.1371/journal.pgph.0005196)*,* [*https://doi.org/10.1371/journal.pgph.0006139*](https://doi.org/10.1371/journal.pgph.0006139)*,* [*https://doi.org/10.1371/journal.pgph.0004997*](https://doi.org/10.1371/journal.pgph.0004997)*,* [*https://doi.org/10.1371/journal.pgph.0004225*](https://doi.org/10.1371/journal.pgph.0004225)*,* [*https://doi.org/10.1371/journal.pgph.0004880*](https://doi.org/10.1371/journal.pgph.0004880)*].*

*Our PRISMA 2020-compliant flow diagram (Figure 1) documents the complete screening process, including the number of studies excluded at each stage and the primary reasons for exclusion at the full-text stage (not construction-related, n=464; no migrant vs. non-migrant comparison, n=1,394; no occupational health outcome, n=67; no sample size reported, n=2; published before 1990, n=3; other, n=5). We believe this satisfies the spirit of this requirement and is consistent with the journal's existing publication standards.*

5.3. If any of the included studies are unpublished, include a link (URL) to the primary source or detailed information about how the content can be accessed.

*Point 5.3 (unpublished studies): All 38 included studies are published in peer-reviewed journals or as grey literature reports, with full citations provided in the reference list.*

5.4. A table of all data extracted from the primary research sources for the systematic review and/or meta-analysis. The table must include the following information for each study:
     5.4.1. Name of data extractors and date of data extraction
     5.4.2. Confirmation that the study was eligible to be included in the review.
     5.4.3. All data extracted from each study for the reported systematic review and/or meta-analysis that would be needed to replicate your analyses.
     5.4.4. If data or supporting information were obtained from another source (e.g. correspondence with the author of the original research article), please provide the source of data and dates on which the data/information were obtained by your research group.

*Points within 5.4 (data extraction table): The data extracted from all 38 included studies are fully reported in the manuscript and supporting information files. Table 1 summarizes the study characteristics including study design, country, study period, population, outcomes, sample sizes, migrant definitions, and quality assessment scores. Tables 2-5 report the extracted outcome data and measures of effect used in synthesis for this review.*

*Data extraction was conducted by two independent reviewers from the following author team: JM and HS then JP and SC during the period 2012 - 2025. JP independently reviewed all extracted data from earlier years for consistency and accuracy. Discrepancies were resolved by discussion, with a third reviewer (HS) consulted where necessary. All included studies were identified through the systematic search. Although study authors were contacted where outcome data were unclear or unavailable, no additional data were obtained via author correspondence.*

*We have added the following statement regarding data extraction to our data analysis section (lines 174-175):*

*Data extraction was conducted in two phases by independent reviewer pairs (JM and HS; JP and SC), with HS serving as arbitrator to resolve discrepancies.*

5.5. If applicable for your analysis, a table showing the completed risk of bias and quality/certainty assessments for each study or outcome. Please ensure this is provided for each domain or parameter assessed. For example, if you used the Cochrane risk-of-bias tool for randomized trials, provide answers to each of the signalling questions for each study. If you used GRADE to assess certainty of evidence, provide judgements about each of the quality of evidence factor. This should be provided for each outcome.

*Point 5.5 (risk of bias): We have added a supplementary table (S1 Table) providing the appraisal responses for each of the 38 included studies, including scores for each individual checklist item rather than aggregate scores only.*

5.6. An explanation of how missing data were handled.

*Point 5.6 (missing data): We have added the following statement to the Methods section (see lines 160-163):*

*Where authors did not respond or data remained unavailable, studies were retained in the narrative synthesis if they continued to meet the inclusion criteria. For example, studies reporting prevalence data without effect measures were still included in our review for descriptive statistics purposes only.*

6. If the reviewer comments include a recommendation to cite specific previously published works, please review and evaluate these publications to determine whether they are relevant and should be cited. There is no requirement to cite these works unless the editor has indicated otherwise.

*The reviewer below has not recommended to cite any specific previously published works.*

**Additional Editor Comments (if provided):**


**Reviewers' comments:**

Reviewer's Responses to Questions

**Comments to the Author**

1. Does this manuscript meet PLOS Global Public Health’s [publication criteria](https://track.editorialmanager.com/CL0/https:%2F%2Fjournals.plos.org%2Fglobalpublichealth%2Fs%2Fcriteria-for-publication/1/010f019ddc6ca2b0-5dffaffa-e208-495a-a043-5c89c6a8da12-000000/lrmzabeDQDL0aItJRSx7MCdOetNNopWfPjdiZUMechE=258)? Is the manuscript technically sound, and do the data support the conclusions? The manuscript must describe methodologically and ethically rigorous research with conclusions that are appropriately drawn based on the data presented.

Reviewer #1: Yes

2. Has the statistical analysis been performed appropriately and rigorously?

Reviewer #1: N/A

3. Have the authors made all data underlying the findings in their manuscript fully available (please refer to the Data Availability Statement at the start of the manuscript PDF file)?

The [PLOS Data policy](https://track.editorialmanager.com/CL0/https:%2F%2Fjournals.plos.org%2Fglobalpublichealth%2Fs%2Fdata-availability/1/010f019ddc6ca2b0-5dffaffa-e208-495a-a043-5c89c6a8da12-000000/ioRJoHv1vCwRR1nwxYC1zx_fwNrKpfJAwU8GIAJGKSE=258) requires authors to make all data underlying the findings described in their manuscript fully available without restriction, with rare exception. The data should be provided as part of the manuscript or its supporting information, or deposited to a public repository. For example, in addition to summary statistics, the data points behind means, medians and variance measures should be available. If there are restrictions on publicly sharing data—e.g. participant privacy or use of data from a third party—those must be specified.

Reviewer #1: Yes

4. Is the manuscript presented in an intelligible fashion and written in standard English?

PLOS Global Public Health does not copyedit accepted manuscripts, so the language in submitted articles must be clear, correct, and unambiguous. Any typographical or grammatical errors should be corrected at revision, so please note any specific errors here.

Reviewer #1: Yes

5. Review Comments to the Author

Please use the space provided to explain your answers to the questions above. You may also include additional comments for the author, including concerns about dual publication, research ethics, or publication ethics. (Please upload your review as an attachment if it exceeds 20,000 characters)

**Reviewer #1:** This is an interesting systematic review. I have a few minor suggestions:

-In the abstract where you mention: "Several studies examined associations between adverse health outcomes and socio-demographic characteristics like age, gender, and ethnicity; language barriers; psychosocial and cultural factors; years of employment and work experience; and access to comprehensive safety training and adequate protective gear." It is not clear if these were the factors that came out to be significantly associated with morbidity or mortality or were these only examined in the studies? Please clarify that. Moreover, I would recommend focusing on occupational factors that were associated (like years of employment, etc.)

We thank the reviewer for their suggestion. We have amended the sentence in the abstract to clarify this point and focus on factors that were associated with adverse health outcomes. This sentence has been amended as follows (see lines 47-53):

*Several occupational and structural factors were significantly associated with morbidity and/or mortality among migrant construction workers, including fewer years of employment and work experience, type of work (e.g., skilled vs. unskilled roles), limited safety training and attendance of briefings, and lower educational attainment. Socio-demographic and cultural factors were also associated with adverse health outcomes, including poor language proficiency, ethnicity, gender, and risk-taking attitudes and low awareness of workplace hazards; the role of age varied across studies.*

-In your discussion section, can you describe if any differences were identified between HICs vs LMICs. I know there were only few studies from LMICs, but that would be relevant to add in discussion.

We thank the reviewer for this suggestion. Upon reflection, we determined that drawing meaningful comparisons between high-income and low- and middle-income country (LMIC) settings was not feasible given that only one study was conducted in a LMIC (Nuewayhid et al., Lebanon). We have therefore chosen to address this point by adding to our limitation section to more explicitly acknowledge the underrepresentation of LMIC settings, rather than risk overstating what a single study can support. The revised text reads as follows (see lines 543-546):

*Furthermore, the majority of included studies were conducted in high income countries (n=35), with three studies from upper-middle income settings (Iran, China, Thailand) and only one from a lower-middle income country (Lebanon), precluding meaningful comparisons across income settings.*

-Finally, in discussion, can you comment on the methodological aspects (study design, sample size, sampling approach, robust analysis) of the studies and if you consider there were limitations in these aspects.

We thank the reviewer for this comment. While methodological limitations relating to study design variability (lines 550-554), sampling and selection bias (lines 538-543, 555-565), and comparability constraints (lines 550-554, 566-568) were already discussed, we have further expanded the limitations section to explicitly address limitations related to sampling approach, sample size, and confounder adjustment(lines 568- 580):

*Given the limited evidence base, we adopted broad inclusion criteria and did not exclude studies on the basis of appraised quality; as a result, some lower-quality studies were included. In addition, many studies relied on non-probability sampling (e.g., purposive or convenience samples) and had small sample sizes, particularly those conducted in hospital and construction- site settings, limiting statistical power and generalizability; the larger registry-and cohort-based studies were in turn constrained by the limitations of administrative data. Many studies also lacked adjustment for key confounders including job tenure, firm size, socioeconomic status, language proficiency, undocumented status, type of construction work, and length of time in host country, reducing the robustness of reported effect estimates and limiting the attribution of observed disparities specifically to migrant status. Collectively, these limitations underscore the need for rigorous research directly comparing outcomes between migrant and non-migrant construction workers to inform future resource allocation and policy.*

-In section of future research: please highlight the need for high-quality interventional studies (including trials) to identify low-cost solutions to address the occupational morbidity and mortality among migrant construction workers.

We thank the reviewer for this suggestion. We have added the following text to the future research section of our discussion (see lines 661-664):

*Ultimately, high-quality interventional research, including pragmatic trials and quasi-experimental studies, is urgently needed to identify low-cost, scalable solutions for reducing occupational morbidity and mortality among migrant construction workers.*

6. PLOS authors have the option to publish the peer review history of their article ([what does this mean?](https://track.editorialmanager.com/CL0/https:%2F%2Fjournals.plos.org%2Fglobalpublichealth%2Fs%2Feditorial-and-peer-review-process%23loc-peer-review-history/1/010f019ddc6ca2b0-5dffaffa-e208-495a-a043-5c89c6a8da12-000000/d6d_eIV-ik5KG8vSheL9sHfXCDAxsCC_E5Suj3wVj_w=258)). If published, this will include your full peer review and any attached files.


**Do you want your identity to be public for this peer review?** If you choose “no”, your identity will remain anonymous but your review may still be made public.


For information about this choice, including consent withdrawal, please see our [Privacy Policy](https://track.editorialmanager.com/CL0/https:%2F%2Fwww.plos.org%2Fprivacy-policy/1/010f019ddc6ca2b0-5dffaffa-e208-495a-a043-5c89c6a8da12-000000/_LiBfPVvN_nbFcjJ96qegUmdtziZac8HuufGMfuVB5w=258).

Reviewer #1: **Yes:** Asaad Ahmed Nafees

[NOTE: If reviewer comments were submitted as an attachment file, they will be attached to this email and accessible via the submission site. Please log into your account, locate the manuscript record, and check for the action link "View Attachments". If this link does not appear, there are no attachment files.]

**Figure Resubmissions:**

While revising your submission, we strongly recommend that you use PLOS’s NAAS tool (<https://ngplosjournals.pagemajik.ai/artanalysis>) to test your figure files. NAAS can convert your figure files to the TIFF file type and meet basic requirements (such as print size, resolution), or provide you with a report on issues that do not meet our requirements and that NAAS cannot fix.

After uploading your figures to PLOS’s NAAS tool - <https://ngplosjournals.pagemajik.ai/artanalysis>, NAAS will process the files provided and display the results in the "Uploaded Files" section of the page as the processing is complete. If the uploaded figures meet our requirements (or NAAS is able to fix the files to meet our requirements), the figure will be marked as "fixed" above. If NAAS is unable to fix the files, a red "failed" label will appear above. When NAAS has confirmed that the figure files meet our requirements, please download the file via the download option, and include these NAAS processed figure files when submitting your revised manuscript.
