# Supplementary material for: Occupational health outcomes among migrant and non-migrant construction workers: A systematic review and narrative synthesis
Source: PLOS Glob Public Health. 2026 Nov 13;6(11):e0007624. doi: 10.1371/journal.pgph.0007624 (PMC13766298; doi:10.1371/journal.pgph.0007624)
Supplement: Supplementary file 6 [file pgph.0007624.s008.docx]

**Journal Requirements:**

If the reviewer comments include a recommendation to cite specific previously published works, please review and evaluate these publications to determine whether they are relevant and should be cited. There is no requirement to cite these works unless the editor has indicated otherwise.

Please review your reference list to ensure that it is complete and correct. If you have cited papers that have been retracted, please include the rationale for doing so in the manuscript text, or remove these references and replace them with relevant current references. Any changes to the reference list should be mentioned in the rebuttal letter that accompanies your revised manuscript. If you need to cite a retracted article, indicate the article’s retracted status in the References list and also include a citation and full reference for the retraction notice.
*Thank you for these reminders, we have made sure to fulfill these requirements in our submission.*

**Additional Editor Comments (if provided):**

Reviewer 2:

This is an interesting systematic review on a very important topic. The methodology is robust, the results are clear although quite descriptive.

I have several minor comments:
1. Throughout the results, and in particular in the sub-section on non-fatal injuries, the authors show how different studies, even if they were conducted in the same country, report very different results. While some studies show that migrant workers are at higher risk than native workers, others show the opposite results. Authors touch upon this in the discussion section but I think it would be helpful to already add a comment regarding this in the results, why there are those differences - different methodologies? Different data sets (you mentioned that most studies work with national data)? Different years of research or focus on different population groups?
*Thank you for your feedback. In addition to what we touch upon in the discussion section, we have added a paragraph at the end of the 'Non-Fatal Injuries' Results section addressing differences in data sources, sampled populations, and study periods, and how these may explain the contrasting findings (Lines 278-301):*

*“Differences in data sources, sampled populations, and study periods may partially explain the variation in findings within and across countries. In Italy, for example, studies using national administrative registry data from the private sector (Giraudo et al. 2017, data sourced from 2000 to 2005; Giraudo et al. 2019, data sourced from 2005 and 2010) and a national labour force survey (Salvatore et al., data sourced from 2007) identified elevated injury risk among certain groups of migrants, while a survey of trainee workers at a single vocational school (Ricci et al., published in 2021, data collection period not reported) found lower odds of reported injury among Eastern European and Balkan migrants compared to local Italian workers (35, 41, 42, 64). Similarly, in the United States, a single-site study conducted at a metropolitan trauma hospital (Forst et al., qualitative interviews conducted in 2003) found higher counts of injuries among foreign-born workers, whereas a national household survey (Zhang et al., data sourced from 1997 to 2005) identified higher rates of injury among US-born workers (37, 56). In South Korea, a national compensation registry study (Cha and Cho, data sourced from 2005-2007) identified elevated non-fatal injury risk among migrants, while a survey of workers at selected construction sites conducted approximately 10 years later (Korkmaz and Park, data sourced from 2016) found no significant difference in injuries between foreign workers and Korean workers (28, 60). More information regarding study design and population is detailed in Table 1.*

*These contrasting findings are consistent with differences in ascertainment: registry- and survey-based studies capture formally reported injuries relative to workforce denominators, whereas hospital-based series and self-report samples reflect care seeking, reporting behavior, and workforce composition at a given site, each of which may differ between migrant and non-migrant workers; the site- and school-based surveys additionally relied on non-random samples. Temporal factors, including economic cycles and changes in migrant workforce composition and safety regulation, may further contribute to divergent results across study periods.”*

2. In the sub-section on “Other Factors Associated with Adverse Occupational Health Outcomes”, I would suggest to make the reporting on suicide as a stand alone paragraph. Its currently part of the paragraph reporting on language proficiency. I think it is important to separate the two discussions and highlight suicide as a separate crucial point of research.

*Thank you for this suggestion. King et al.’s findings regarding suicide are discussed in two sections of the manuscript. First, in the “Morbidity” section within the psychological illness paragraph, suicide rate is presented as a general comparative outcome between native and foreign national construction workers from Australia vs. English vs. non-English speaking countries (Lines 434-437). Second, in the “Other Factors Associated with Adverse Occupational Health Outcomes” within the language proficiency paragraph, suicide rates are revisited to highlight language proficiency as a potential moderating factor, given suicide rates varied between workers from English-speaking and non-English speaking countries of origin from 2006 to 2020 (Lines 545-553). We believe the current placement reflects the thematic organization of the manuscript.*

3. In the recommendations section of the article, authors suggest the following - “Analyses directly comparing outcomes between migrant and non-migrant workers, disaggregated by demographic characteristics like gender and ethnicity and mechanisms of injury, will additionally enable rigorous meta-analytic estimates and demographically responsive analysis and policy development.” I am not convinced by the recommendation to segregate data by ethnicity as it is a very challenging characteristic and does not necessarily indicate migration status. Instead, I would suggest focusing on the country of birth.

*Thank you for this helpful point. We agree that ethnicity does not indicate migration status and we have added “country of birth,” to the demographic characteristics listed. We have kept “ethnicity” as a complementary characteristic since it conveys how workers’ language, cultural practices, and ethnic identity/affiliation may influence occupational health outcomes, that are not fully captured by country of birth alone, as observed in several included studies (e.g., Ricci et al., Capasso et al.) (Lines 760-763):*

*“Analyses directly comparing outcomes between migrant and non-migrant workers, disaggregated by demographic characteristics like gender, country of birth, ethnicity, and mechanisms of injury, will additionally enable rigorous meta-analytic estimates and demographically responsive analysis and policy development. “*

4. In the methodology section, authors indicated that they collected results for both adults and children labour migrants. However, I did not see a discussion on child labourers in the results, were there any studies focusing on this population?

*Thank you for pointing this out. Our eligibility criteria placed no restriction on age, so studies involving child construction workers were eligible for inclusion. While a few studies mentioned workers younger than 18 years old, no included study examined or reported outcomes for child migrant and non-migrant construction workers as distinct populations. We have added a note to the Future Research section of the Discussion flagging children engaged in construction work as an understudied group (Lines 769-776):*

*“While a small number of included studies noted workers as young as sixteen or seventeen years old in their sample, no study examined child construction workers as a distinct population or reported outcomes disaggregated for migrant and non-migrant construction workers under 18. Several studies also did not report participant age, meaning some samples may have included minors. Children engaged in construction work therefore remain an understudied and potentially highly vulnerable group, and future studies should report participant age and examine outcomes for workers under 18.”*

5. Page 50, lines 224-226 - “In Italy, for example, Elders et al. (52) found that Turkish scaffolders faced over twice the risk of disability compared to Dutch scaffolders (risk ratio [RR] 2.48, 95% CI 1.94-3.18), even after adjusting for age.” - there seem to be a mistake in the sentence, should it be "In the Netherlands" instead of "In Italy"?

*Thank you for identifying this error. Elders et al. (2004) was conducted in the Netherlands, not Italy. We have corrected this in the revised manuscript (Line 241).*

6. Page 57, lines 297-300 - “In Ireland, Connell et al. reported similar counts but greater severity (requiring surgical intervention) among non-migrants (80 Irish, 75 total migrants [45 Polish, 5 Romanian, 16 Lithuanian, 9 Other] reports of construction-related ocular injuries; 0 vs. 8 penetrating ocular injuries, in Irish and non-nationals respectively) (50). - unclear sentence structure.” - the sentence is quite confusing to read with different types of brackets.

*Thank you for this feedback. We have revised the sentence to improve clarity (Lines 385-390):*

*“In Ireland, Connell et al. reported comparable total counts of construction-related ocular injuries between Irish nationals (n=80) and non-national workers (n=75, comprised of 45 Polish, 5 Romanian, 16 Lithuanian, and 9 other workers), but greater severity of injuries among non-nationals. Specifically, all eight cases of penetrating ocular injury required surgical intervention and occurred in non-national workers, compared to none among Irish workers (50).”*
