# Supplementary material for: Occupational health outcomes among migrant and non-migrant construction workers: A systematic review and narrative synthesis
Source: PLOS Glob Public Health. 2026 Nov 13;6(11):e0007624. doi: 10.1371/journal.pgph.0007624 (PMC13766298; doi:10.1371/journal.pgph.0007624)
Supplement: Peer review history

# Peer review: Decision Letter 0

**DOI:** 10.1371/journal.pgph.0007624.r001

**Authors:** Emma Campbell

29 Apr 2026

PGPH-D-26-00534

Occupational health outcomes among migrant and non-migrant construction workers: a systematic review and narrative synthesis

PLOS Global Public Health

Dear Dr. Buller,

Thank you for submitting your manuscript to PLOS Global Public Health. After careful consideration, we feel that it has merit but does not fully meet PLOS Global Public Health’s publication criteria as it currently stands. Therefore, we invite you to submit a revised version of the manuscript that addresses the points raised during the review process.

Please note that we have only been able to secure a single reviewer to assess your manuscript. We are issuing a decision on your manuscript at this point to prevent further delays in the evaluation of your manuscript. Please be aware that the editor who handles your revised manuscript might find it necessary to invite additional reviewers to assess this work once the revised manuscript is submitted. However, we will aim to proceed on the basis of this single review if possible.

Please review their comments and make the appropriate changes to the manuscript.

Please submit your revised manuscript by Jun 28 2026 11:59PM. If you will need more time than this to complete your revisions, please reply to this message or contact the journal office at globalpubhealth@plos.org. When you're ready to submit your revision, log on to https://www.editorialmanager.com/pgph/ and select the 'Submissions Needing Revision' folder to locate your manuscript file.

Please include the following items when submitting your revised manuscript:

- A letter that responds to each point raised by the editor and reviewer(s). You should upload this letter as a separate file labeled 'Response to Reviewers'.
- A marked-up copy of your manuscript that highlights changes made to the original version. You should upload this as a separate file labeled 'Revised Manuscript with Track Changes'.
- An unmarked version of your revised paper without tracked changes. You should upload this as a separate file labeled 'Manuscript'.

Guidelines for resubmitting your figure files are available below the reviewer comments at the end of this letter.

As the corresponding author, your ORCID iD is verified in the submission system and will appear in the published article. PLOS supports the use of ORCID, and we encourage all coauthors to register for an ORCID iD and use it as well. Please encourage your coauthors to verify their ORCID iD within the submission system before final acceptance, as unverified ORCID iDs will not appear in the published article. *Only*  the individual author can complete the verification step; PLOS staff *cannot*  verify ORCID iDs on behalf of authors.

We look forward to receiving your revised manuscript.

Kind regards,

Emma Campbell, Ph.D

Staff Editor

PLOS Global Public Health

**Journal Requirements:**

1. Please amend your detailed Financial Disclosure statement. This is published with the article. It must therefore be completed in full sentences and contain the exact wording you wish to be published.

i. Please clarify all sources of financial support for your study. List the grants, grant numbers, and organizations that funded your study, including funding received from your institution. Please note that suppliers of material support, including research materials, should be recognized in the Acknowledgements section rather than in the Financial Disclosure.

ii. State the initials, alongside each funding source, of each author to receive each grant. For example: "This work was supported by the National Institutes of Health (####### to AM; ###### to CJ) and the National Science Foundation (###### to AM)."

iii. State what role the funders took in the study. If the funders had no role in your study, please state: “The funders had no role in study design, data collection and analysis, decision to publish, or preparation of the manuscript.”

iv. If any authors received a salary from any of your funders, please state which authors and which funders.

2. We note that your Data Availability Statement is currently as follows: All relevant data regarding the systematic review is including within the paper and Supporting Information files.

Please confirm at this time whether or not your submission contains all raw data required to replicate the results of your study. Authors must share the “minimal data set” for their submission. PLOS defines the minimal data set to consist of the data required to replicate all study findings reported in the article, as well as related metadata and methods (https://journals.plos.org/plosone/s/data-availability#loc-minimal-data-set-definition).

For example, authors should submit the following data:

- The values behind the means, standard deviations and other measures reported;

- The values used to build graphs;

- The points extracted from images for analysis.

Authors do not need to submit their entire data set if only a portion of the data was used in the reported study.

If your submission does not contain these data, please either upload them as Supporting Information files or deposit them to a stable, public repository and provide us with the relevant URLs, DOIs, or accession numbers. For a list of recommended repositories, please see https://journals.plos.org/plosone/s/recommended-repositories.

If there are ethical or legal restrictions on sharing a de-identified data set, please explain them in detail (e.g., data contain potentially sensitive information, data are owned by a third-party organization, etc.) and who has imposed them (e.g., an ethics committee). Please also provide contact information for a data access committee, ethics committee, or other institutional body to which data requests may be sent. If data are owned by a third party, please indicate how others may request data access.

3. Please provide separate figure files in .tif or .eps format.

For more information about figure files please see our guidelines:

https://journals.plos.org/globalpublichealth/s/figures

https://journals.plos.org/globalpublichealth/s/figures#loc-file-requirements

4. We have noticed that you have uploaded Supporting Information files, but you have not included a list of legends. Please add a full list of legends for your Supporting Information files after the references list.

5. As required by our policy on Data Availability, please ensure your manuscript or supplementary information includes the following:

A numbered table of all studies identified in the literature search, including those that were excluded from the analyses.

For every excluded study, the table should list the reason(s) for exclusion.

If any of the included studies are unpublished, include a link (URL) to the primary source or detailed information about how the content can be accessed.

A table of all data extracted from the primary research sources for the systematic review and/or meta-analysis. The table must include the following information for each study:

Name of data extractors and date of data extraction

Confirmation that the study was eligible to be included in the review.

All data extracted from each study for the reported systematic review and/or meta-analysis that would be needed to replicate your analyses.

If data or supporting information were obtained from another source (e.g. correspondence with the author of the original research article), please provide the source of data and dates on which the data/information were obtained by your research group.

If applicable for your analysis, a table showing the completed risk of bias and quality/certainty assessments for each study or outcome.  Please ensure this is provided for each domain or parameter assessed. For example, if you used the Cochrane risk-of-bias tool for randomized trials, provide answers to each of the signalling questions for each study. If you used GRADE to assess certainty of evidence, provide judgements about each of the quality of evidence factor. This should be provided for each outcome.

An explanation of how missing data were handled.

This information can be included in the main text, supplementary information, or relevant data repository. Please note that providing these underlying data is a requirement for publication in this journal, and if these data are not provided your manuscript might be rejected.

If the reviewer comments include a recommendation to cite specific previously published works, please review and evaluate these publications to determine whether they are relevant and should be cited. There is no requirement to cite these works unless the editor has indicated otherwise.

**Additional Editor Comments (if provided):**

[Note: HTML markup is below. Please do not edit.]

Reviewers' comments:

Reviewer's Responses to Questions

**Comments to the Author**

1. Does this manuscript meet PLOS Global Public Health’s publication criteria?

Reviewer #1: Yes

**********

2. Has the statistical analysis been performed appropriately and rigorously?

Reviewer #1: N/A

**********

3. Have the authors made all data underlying the findings in their manuscript fully available (please refer to the Data Availability Statement at the start of the manuscript PDF file)?

The PLOS Data policy

Reviewer #1: Yes

**********

4. Is the manuscript presented in an intelligible fashion and written in standard English?

Reviewer #1: Yes

**********

**Reviewer #1:**  This is an interesting systematic review. I have a few minor suggestions:

-In the abstract where you mention: "Several studies examined associations between adverse health outcomes and socio-demographic characteristics like age, gender, and ethnicity; language barriers; psychosocial and cultural factors; years of employment and work experience; and access to comprehensive safety training and adequate protective gear." It is not clear if these were the factors that came out to be significantly associated with morbidity or mortality or were these only examined in the studies? Please clarify that. Moreover, I would recommend focusing on occupational factors that were associated (like years of employment, etc.)

-In your discussion section, can you describe if any differences were identified between HICs vs LMICs. I know there were only few studies from LMICs, but that would be relevant to add in discussion.

-Finally, in discussion, can you comment on the methodological aspects (study design, sample size, sampling approach, robust analysis) of the studies and if you consider there were limitations in these aspects.

-In section of future research: please highlight the need for high-quality interventional studies (including trials) to identify low-cost solutions to address the occupational morbidity and mortality among migrant construction workers.

**********

what does this mean?). If published, this will include your full peer review and any attached files.

**Do you want your identity to be public for this peer review?**  If you choose “no”, your identity will remain anonymous but your review may still be made public.

For information about this choice, including consent withdrawal, please see our Privacy Policy

Reviewer #1: **Yes:**  Asaad Ahmed Nafees

**********

[NOTE: If reviewer comments were submitted as an attachment file, they will be attached to this email and accessible via the submission site. Please log into your account, locate the manuscript record, and check for the action link "View Attachments". If this link does not appear, there are no attachment files.]

**Figure Resubmissions:**

# Author comment: Author response to Decision Letter 1

**DOI:** 10.1371/journal.pgph.0007624.r002

11 Jun 2026

Attachment Submitted filename: Response to Reviewers.docx

# Peer review: Decision Letter 1

**DOI:** 10.1371/journal.pgph.0007624.r003

**Authors:** Priyanka Roy

18 Aug 2026

PGPH-D-26-00534R1

Occupational health outcomes among migrant and non-migrant construction workers: a systematic review and narrative synthesis

PLOS Global Public Health

Dear Dr. Buller,

Thank you for submitting your manuscript to PLOS Global Public Health. After careful consideration, we feel that it has merit but does not fully meet PLOS Global Public Health’s publication criteria as it currently stands. Therefore, we invite you to submit a revised version of the manuscript that addresses the points raised during the review process.

Please submit your revised manuscript by Sep 17 2026 11:59PM. If you will need more time than this to complete your revisions, please reply to this message or contact the journal office at globalpubhealth@plos.org. When you're ready to submit your revision, log on to https://www.editorialmanager.com/pgph/ and select the 'Submissions Needing Revision' folder to locate your manuscript file.

Please include the following items when submitting your revised manuscript:

- A letter that responds to each point raised by the editor and reviewer(s). You should upload this letter as a separate file labeled 'Response to Reviewers'.
- A marked-up copy of your manuscript that highlights changes made to the original version. You should upload this as a separate file labeled 'Revised Manuscript with Track Changes'.
- An unmarked version of your revised paper without tracked changes. You should upload this as a separate file labeled 'Manuscript'.

Guidelines for resubmitting your figure files are available below the reviewer comments at the end of this letter.

As the corresponding author, your ORCID iD is verified in the submission system and will appear in the published article. PLOS supports the use of ORCID, and we encourage all coauthors to register for an ORCID iD and use it as well. Please encourage your coauthors to verify their ORCID iD within the submission system before final acceptance, as unverified ORCID iDs will not appear in the published article. *Only*  the individual author can complete the verification step; PLOS staff *cannot*  verify ORCID iDs on behalf of authors.

We look forward to receiving your revised manuscript.

Kind regards,

Priyanka Roy, MD, MPH, PhD

Academic Editor

PLOS Global Public Health

Journal Requirements:

If the reviewer comments include a recommendation to cite specific previously published works, please review and evaluate these publications to determine whether they are relevant and should be cited. There is no requirement to cite these works unless the editor has indicated otherwise.

Please review your reference list to ensure that it is complete and correct. If you have cited papers that have been retracted, please include the rationale for doing so in the manuscript text, or remove these references and replace them with relevant current references. Any changes to the reference list should be mentioned in the rebuttal letter that accompanies your revised manuscript. If you need to cite a retracted article, indicate the article’s retracted status in the References list and also include a citation and full reference for the retraction notice.

Additional Editor Comments (if provided):

Reviewer 2:

This is an interesting systematic review on a very important topic. The methodology is robust, the results are clear although quite descriptive.

I have several minor comments:

1. Throughout the results, and in particular in the sub-section on non-fatal injuries, the authors show how different studies, even if they were conducted in the same country, report very different results. While some studies show that migrant workers are at higher risk than native workers, others show the opposite results. Authors touch upon this in the discussion section but I think it would be helpful to already add a comment regarding this in the results, why there are those differences - different methodologies? Different data sets (you mentioned that most studies work with national data)? Different years of research or focus on different population groups?

2. In the sub-section on “Other Factors Associated with Adverse Occupational Health Outcomes”, I would suggest to make the reporting on suicide as a stand alone paragraph. Its s currently part of the paragraph reporting on language proficiency. I think it is important to separate the two discussions and highlight suicide as a separate crucial point of research.

3. In the recommendations section of the article, authors suggest the following - “Analyses directly comparing outcomes between migrant and non-migrant workers, disaggregated by demographic characteristics like gender and ethnicity and mechanisms of injury, will additionally enable rigorous meta-analytic estimates and demographically responsive analysis and policy development.” I am not convinced by the recommendation to segregate data by ethnicity as it is a very challenging characteristic and does not necessarily indicate migration status. Instead, I would suggest focusing on the country of birth.

4. In the methodology section, authors indicated that they collected results for both adults and children labour migrants. However, I did not see a discussion on child labourers in the results, were there any studies focusing on this population?

5. Page 50, lines 224-226 - “In Italy, for example, Elders et al. (52) found that Turkish scaffolders faced over twice the risk of disability compared to Dutch scaffolders (risk ratio [RR] 2.48, 95% CI 1.94-3.18), even after adjusting for age.” - there seem to be a mistake in the sentence, should it be "In the Netherlands" instead of "In Italy"?

6. Page 57, lines 297-300 - “In Ireland, Connell et al. reported similar counts but greater severity (requiring surgical intervention) among non-migrants (80 Irish, 75 total migrants [45 Polish, 5 Romanian, 16 Lithuanian, 9 Other] reports of construction-related ocular injuries; 0 vs. 8 penetrating ocular injuries, in Irish and non-nationals respectively) (50). - unclear sentence structure.” - the sentence is quite confusing to read with different types of brackets.

[Note: HTML markup is below. Please do not edit.]

Reviewers' comments:

Reviewer's Responses to Questions

**Comments to the Author**

Reviewer #2: (No Response)

**********

publication criteria?

Reviewer #2: Yes

**********

3. Has the statistical analysis been performed appropriately and rigorously?

Reviewer #2: N/A

**********

4. Have the authors made all data underlying the findings in their manuscript fully available (please refer to the Data Availability Statement at the start of the manuscript PDF file)?

The PLOS Data policy

Reviewer #2: Yes

**********

5. Is the manuscript presented in an intelligible fashion and written in standard English?

Reviewer #2: Yes

**********

Reviewer #2: This is an interesting systematic review on a very important topic. The methodology is robust, the results are clear although quite descriptive.

I have several minor comments:

1. Throughout the results, and in particular in the sub-section on non-fatal injuries, the authors show how different studies, even if they were conducted in the same country, report very different results. While some studies show that migrant workers are at higher risk than native workers, others show the opposite results. Authors touch upon this in the discussion section but I think it would be helpful to already add a comment regarding this in the results, why there are those differences - different methodologies? Different data sets (you mentioned that most studies work with national data)? Different years of research or focus on different population groups?

2. In the sub-section on “Other Factors Associated with Adverse Occupational Health Outcomes”, I would suggest to make the reporting on suicide as a stand alone paragraph. Its s currently part of the paragraph reporting on language proficiency. I think it is important to separate the two discussions and highlight suicide as a separate crucial point of research.

3. In the recommendations section of the article, authors suggest the following - “Analyses directly comparing outcomes between migrant and non-migrant workers, disaggregated by demographic characteristics like gender and ethnicity and mechanisms of injury, will additionally enable rigorous meta-analytic estimates and demographically responsive analysis and policy development.” I am not convinced by the recommendation to segregate data by ethnicity as it is a very challenging characteristic and does not necessarily indicate migration status. Instead, I would suggest focusing on the country of birth.

4. In the methodology section, authors indicated that they collected results for both adults and children labour migrants. However, I did not see a discussion on child labourers in the results, were there any studies focusing on this population?

5. Page 50, lines 224-226 - “In Italy, for example, Elders et al. (52) found that Turkish scaffolders faced over twice the risk of disability compared to Dutch scaffolders (risk ratio [RR] 2.48, 95% CI 1.94-3.18), even after adjusting for age.” - there seem to be a mistake in the sentence, should it be "In the Netherlands" instead of "In Italy"?

6. Page 57, lines 297-300 - “In Ireland, Connell et al. reported similar counts but greater severity (requiring surgical intervention) among non-migrants (80 Irish, 75 total migrants [45 Polish, 5 Romanian, 16 Lithuanian, 9 Other] reports of construction-related ocular injuries; 0 vs. 8 penetrating ocular injuries, in Irish and non-nationals respectively) (50). - unclear sentence structure.” - the sentence is quite confusing to read with different types of brackets.

**********

what does this mean?). If published, this will include your full peer review and any attached files.

**Do you want your identity to be public for this peer review?**  If you choose “no”, your identity will remain anonymous but your review may still be made public.

For information about this choice, including consent withdrawal, please see our Privacy Policy

Reviewer #2: No

**********

[NOTE: If reviewer comments were submitted as an attachment file, they will be attached to this email and accessible via the submission site. Please log into your account, locate the manuscript record, and check for the action link "View Attachments". If this link does not appear, there are no attachment files.]

# Author comment: Author response to Decision Letter 2

**DOI:** 10.1371/journal.pgph.0007624.r004

25 Sep 2026

Attachment Submitted filename: Response_to_Reviewers_auresp_2.docx

# Editor report: Decision Letter 2

**DOI:** 10.1371/journal.pgph.0007624.r005

**Authors:** Priyanka Roy

29 Oct 2026

Occupational health outcomes among migrant and non-migrant construction workers: a systematic review and narrative synthesis

PGPH-D-26-00534R2

Dear Dr. Buller,

We are pleased to inform you that your manuscript 'Occupational health outcomes among migrant and non-migrant construction workers: a systematic review and narrative synthesis' has been provisionally accepted for publication in PLOS Global Public Health.

Before your manuscript can be formally accepted you will need to complete some formatting changes, which you will receive in a follow up email. A member of our team will be in touch with a set of requests.

Please note that your manuscript will not be scheduled for publication until you have made the required changes, so a swift response is appreciated.

IMPORTANT: The editorial review process is now complete. PLOS will only permit corrections to spelling, formatting or significant scientific errors from this point onwards. Requests for major changes, or any which affect the scientific understanding of your work, will cause delays to the publication date of your manuscript.

If your institution or institutions have a press office, please notify them about your upcoming paper to help maximize its impact. If they'll be preparing press materials, please inform our press team as soon as possible -- no later than 48 hours after receiving the formal acceptance. Your manuscript will remain under strict press embargo until 2 pm Eastern Time on the date of publication. For more information, please contact globalpubhealth@plos.org.

Thank you again for supporting Open Access publishing; we are looking forward to publishing your work in PLOS Global Public Health.

Best regards,

Priyanka Roy, MD, MPH, PhD

Academic Editor

PLOS Global Public Health

***********************************************************

Reviewer Comments (if any, and for reference):
